# Identification of GRP78 as a novel host factor that facilitates zoonotic porcine deltacoronavirus internalization and replication via clathrin-mediated endocytosis

**DOI:** 10.1128/jvi.00717-26

**Published:** 2026-07-02

**Authors:** Xinrong Zhou, Konstantin I. Ivanov, Xinna Ge, Xin Guo, Jun Han, Yanhong Chen, Lei Zhou, Yongning Zhang, Deyin Guo, Hanchun Yang

**Affiliations:** 1 National Key Laboratory of Veterinary Public Health Safety, China Agricultural University, College of Veterinary Medicine 34752, Beijing, People's Republic of China; 2 Key Laboratory of Animal Epidemiology of Ministry of Agriculture and Rural Affairs, China Agricultural University, College of Veterinary Medicine 34752, Beijing, People's Republic of China; 3 State Key Laboratory of Respiratory Diseases, Guangzhou Medical University 26468, Guangzhou, People's Republic of China; 4 Guangzhou National Laboratory, Guangzhou International Bio-Island, Guangzhou, People's Republic of China; 5 Faculty of Bioengineering and Bioinformatics, Lomonosov Moscow State University 64935, Moscow, Russia; The Ohio State University, Columbus, Ohio, USA

**Keywords:** porcine deltacoronavirus, host factor, glucose-regulated protein 78 (GRP78), viral entry, clathrin-mediated endocytosis

## Abstract

Porcine deltacoronavirus (PDCoV) infects a wide range of hosts and can spread across species. Moreover, it is capable of infecting pAPN-knockout cell lines and pigs, suggesting that other important host factors facilitate PDCoV infection. To identify these important host factors, in this study, we employed co-immunoprecipitation (Co-IP) combined with liquid chromatography-tandem mass spectrometry (LC-MS/MS) to identify 19 significantly upregulated host membrane proteins that interact with the PDCoV S1 protein. Among these, glucose-regulated protein 78 (GRP78) was found to positively regulate PDCoV attachment and internalization, as validated by knockdown, blocking, and overexpression assays. Affinity assays confirmed a strong interaction between GRP78 protein and PDCoV S1 protein. This regulatory function of GRP78 operates in a pAPN-independent way. Mechanistically, we demonstrated that GRP78 interacts with the PDCoV S1 protein via its substrate-binding domain (SBD) and facilitates viral entry through clathrin-mediated endocytosis. Notably, the role of GRP78 in promoting viral entry and replication has also been applied by other coronaviruses, underscoring its potential as a conserved host factor in coronavirus infection. Together, these findings reveal a novel mechanism of PDCoV-host interaction that centers on GRP78-mediated viral attachment and internalization via clathrin-dependent endocytosis.

**IMPORTANCE:**

Porcine deltacoronavirus (PDCoV) represents a significant zoonotic threat with pandemic potential, exhibiting a broad tissue tropism that underscores its capacity for cross-species spread. Current understanding of PDCoV entry mechanisms, however, remains largely limited to the porcine aminopeptidase N (pAPN), whose knockout fails to fully block infection—highlighting the critical need to identify alternative host entry factors. In this study, we identified the cell membrane protein GRP78 as a novel host factor that binds the C-terminal domain (CTD) of the PDCoV S1 protein via its substrate-binding domain (SBD), thereby mediating viral adsorption and internalization. Furthermore, GRP78 engages clathrin to facilitate viral internalization through endocytosis. Notably, GRP78-mediated entry operates independently of pAPN and demonstrates a degree of broad-spectrum activity relevant to other coronaviruses. Collectively, these findings provide new insights into the early entry mechanisms of PDCoV and identify GRP78 as a potential broad-spectrum target for antiviral intervention.

## INTRODUCTION

Coronaviruses (CoVs) are a group of enveloped, positive-sense, single-stranded RNA viruses with a global distribution (1, 2). They are classified under the family *Coronaviridae* and are divided into four genera: alpha-, beta-, gamma-, and delta-CoV. The beta-CoV genus is of particular research and clinical importance because it includes the most pathogenic human coronaviruses (HCoVs) (2–4). Phylogenetic evidence indicates that most alpha- and beta-CoVs originated in bats or rodents, while birds are the primary reservoir for gamma- and delta-CoVs (2). Throughout evolution, coronaviruses have repeatedly crossed species barriers, with some emerging as significant human pathogens (5–7). To date, a total of nine CoVs have been reported to infect humans. These include three alpha-CoVs (HCoV-229E, HCoV-NL63, and canine coronavirus [CCoV-HuPn-2018]), five beta-CoVs (HCoV-OC43, HCoV-HKU1, severe acute respiratory syndrome coronavirus [SARS-CoV], Middle East respiratory syndrome coronavirus [MERS-CoV], and SARS-CoV-2), and one delta-CoV (PDCoV) (2, 4, 8–10). Notably, SARS-CoV-2, CCoV-HuPn-2018, and PDCoV have emerged within the last 5 years, highlighting the growing threat that CoV infections pose to public health. Furthermore, the zoonotic origin of all known HCoVs underscores the importance of continuous research and surveillance of coronaviruses in animals (2, 11).

The ability of coronaviruses to cross species barriers critically depends on the binding of their spike (S) proteins to receptors on the surface of new host cells. This binding is the first and possibly most important step in the infection process, determining the virus’s host range. The CoV spike protein is a homotrimer in which each protomer consists of two functional subunits: the N-terminal S1 subunit, responsible for receptor binding, and the C-terminal S2 subunit, which mediates membrane fusion (12–14). Cryo-electron microscopy (cryo-EM) analysis of the S protein trimeric structure revealed that the S1 subunit contains four core domains (S1A–S1D), with domains A and B having critical roles in receptor binding (15–17). Several host receptors have been identified for CoV attachment and entry. These include aminopeptidase N (APN) (18, 19), angiotensin-converting enzyme 2 (ACE2) (20), dipeptidyl peptidase 4 (DPP4) (21), carcinoembryonic antigen cell adhesion molecule 1 (CEACAM1) (22), and sialic acid carbohydrates (23). Different coronaviruses utilize different receptors for cell entry. For example, CEACAM1 is the receptor for mouse hepatitis virus (MHV) (24), and DPP4 is the receptor for MERS-CoV (21). ACE2 mediates the attachment of HCoV-NL63, SARS-CoV, and SARS-CoV-2 (25–27). APN is the receptor for several alphacoronaviruses, including HCoV-229E, transmissible gastroenteritis virus (TGEV), and porcine epidemic diarrhea virus (PEDV) (18, 28). In addition to proteinaceous receptors, (acetylated) sialic acid carbohydrates can also function as CoV receptors, expanding the range of surface molecules utilized by coronaviruses for cell attachment and entry (29–31).

Porcine deltacoronavirus (PDCoV) is a significant pathogen in the swine industry, causing severe economic losses due to its association with acute diarrhea and vomiting in piglets. First identified in Hong Kong in 2012, PDCoV has since been reported in multiple countries, raising concerns about its rapid spread and zoonotic potential (8, 32–34). Compared to other porcine coronaviruses, such as porcine epidemic diarrhea virus (PEDV) and transmissible gastroenteritis virus (TGEV), PDCoV exhibits several unique genetic and biological traits. These traits include its ability to rapidly cross species barriers, raising concerns about its potential for interspecies transmission and the resulting implications for public health. Notably, PDCoV was identified in three children with acute undifferentiated febrile illness at a hospital in Haiti in 2021 (8). This finding significantly heightened concerns about the epidemic potential of PDCoV, underscoring the need for increased vigilance regarding this emerging virus.

Although knocking out the known PDCoV receptor, pAPN, significantly reduced PDCoV titers in cultured cells (35, 36), complete inhibition of infection was not achieved. This suggests the existence of pAPN-independent viral entry pathways (36). Additionally, the ability of PDCoV to infect APN-knockout piglets and tissues with low pAPN expression suggests that the virus may use alternative receptors for viral attachment and cell entry (37). Given the growing epidemic potential of PDCoV, identifying these host factors is of paramount importance. Several approaches are available for this task, including genome-wide CRISPR screens (38–41), S1-Fc recombinant protein binding assays with membrane proteins (21), RNAi library screening (42), and comparative transcriptomic profiling of susceptible vs non-susceptible cells (43). It is equally important to identify the mechanisms of PDCoV cell entry among the five well-documented and validated options: phagocytosis (mediated by immunoglobulins), macropinocytosis (involving cytoskeleton rearrangement), clathrin-mediated endocytosis (requiring specific receptors), caveolae-mediated endocytosis (requiring specific receptors), and clathrin/caveolae-independent endocytosis (also requiring specific receptors) (44, 45).

Despite its growing significance, the molecular mechanisms underlying PDCoV pathogenesis and interspecies transmission remain poorly understood. Current research has primarily focused on the structural and functional characterization of individual viral proteins. However, gaps remain in our understanding of how these proteins interact with their host counterparts, especially those involved in virus attachment and cell entry. The objective of this study was to address these gaps by identifying novel host factors that specifically interact with the PDCoV spike protein and by elucidating their functional roles during the early steps of viral entry.

## RESULTS

### Host factors pAPN, GRP78, HSPG2, and CD109 are involved in PDCoV infection

**Fig 1:**
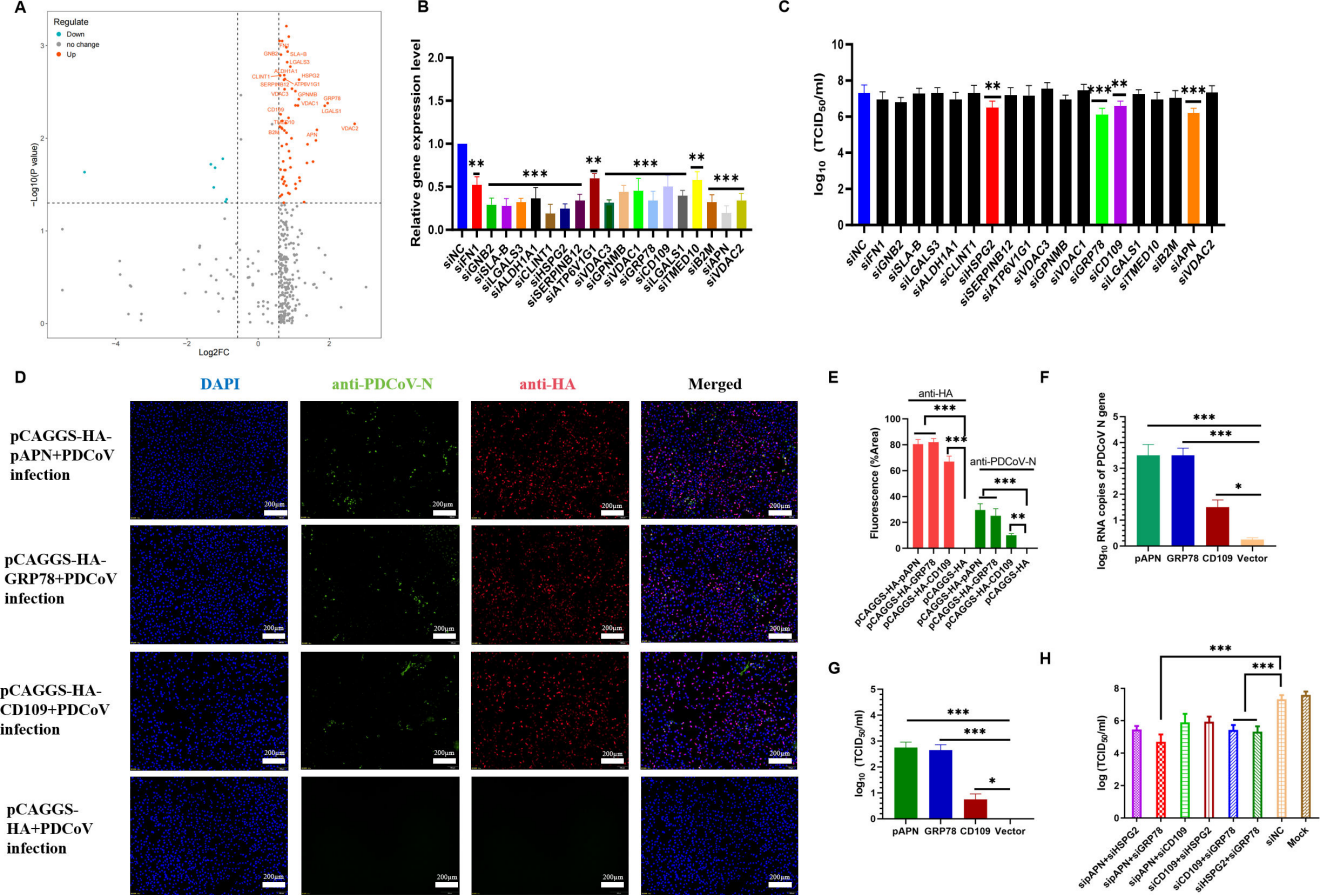
Host factors pAPN, GRP78, HSPG2, and CD109 are involved in PDCoV infection. (**A**) The volcano plot shows the cell membrane proteins identified as potential PDCoV S1 binding partners using a combination of co-immunoprecipitation and mass spectrometry (MS). A total of 19 significantly enriched cell membrane proteins were selected for subsequent functional validation. (**B**) Detection of relative expression levels of target genes after siRNA-specific interference by relative quantitative RT-PCR. (**C**) PDCoV titers in IPEC-J2 cells transfected with siRNAs were measured by the TCID₅₀ assay at 24 hpi. (**D**) Infection intensity of PDCoV in nonpermissive BHK-21 cells overexpressing GRP78, pAPN, or CD109 was measured by IFA. Magnification: 100×. (**E**) Statistical analysis of the fluorescence intensity from multiple images shown in panel D, as measured by ImageJ software. (**F** and **G**) PDCoV infection levels in nonpermissive BHK-21 cells overexpressing GRP78, pAPN, or CD109, as measured by the TCID₅₀ assay and quantitative real-time RT-PCR, respectively. Cells transfected with an empty vector were used as a negative control. (**H**) The titers of PDCoV were examined after two targeted genes knock down simultaneously by siRNA. Data are presented as means ± SD and are representative of at least two independent experiments. Statistical significance is indicated as follows: *P < 0.05, **P < 0.01, ***P < 0.001 (unpaired Student’s *t*-test).

The primary goal of this study was to identify membrane-associated proteins that interact with the PDCoV spike (S) protein, which subsequently facilitate viral infection. To achieve this, we utilized the Co-IP combined with LC-MS/MS to identify the interacting proteins between PDCoV-S1 and the membrane proteins of intestinal porcine epithelial cells (IPEC-J2) derived from the primary tissue targeted by PDCoV. The result of MS was presented in Table S1. A total of 19 membrane proteins with fold change (FC) values (PDCoV-S1 group/Mock group) greater than 1.5 and distribution on the cell surface membrane according to the GeneCard website were selected for subsequent functional experimental validation (Fig. 1A). First, we designed and synthesized small interfering RNAs (siRNAs) specifically targeting these proteins and transfected these siRNAs into IPEC-J2 cells for 24 h. After that, the cells were inoculated with PDCoV at an MOI of 0.1. The results showed that the relative gene expression level of targeted genes was significantly knocked down (Fig. 1B). Among these genes, we observed a significant decrease in PDCoV titers in cells with pAPN, GRP78, HSPG2, and CD109 knockdown (Fig. 1C). These results suggested that these four membrane-associated proteins are involved in PDCoV infection. Second, we transfected the cells with pCAGGS-HA recombinant plasmids expressing HA-tagged pAPN, GRP78, HSPG2, or CD109 to determine whether overexpressing the identified proteins would alter nonpermissive BHK-21 cells susceptible to PDCoV. As shown in Fig. 1D and F, IFA results confirmed the expression of all target proteins in BHK-21 cells except for HSPG2, and the fluorescence intensity was quantified and subsequently analyzed. Among the overexpressed proteins, pAPN and GRP78 had the most significant positive impact on PDCoV infection. The effect of CD109 overexpression was less pronounced but still statistically significant. Furthermore, the results of quantitative real-time RT-PCR (Fig. 1G) and TCID_50_ assays (Fig. 1H) further corroborated this hypothesis, demonstrating a significant increase in PDCoV titers in cells overexpressing pAPN or GRP78. Finally, to determine whether the identified PDCoV S1 binding partners function independently or cooperatively to facilitate PDCoV infection, we performed dual gene silencing experiments in IPEC-J2 cells. We simultaneously silenced two target proteins using their respective siRNAs before infecting the cells with PDCoV. The results of Western blot analysis (Fig. S1A) and quantitative real-time PCR (Fig. S1B) confirmed the efficiency of protein/mRNA were knocked down. As a consequence, dual gene silencing could lead a more significant decrease in PDCoV titers than single gene knockdown (Fig. 1H). These results suggested that the identified membrane-associated proteins may function cooperatively.

### GRP78 is essential for PDCoV attachment and internalization

**Fig 2:**
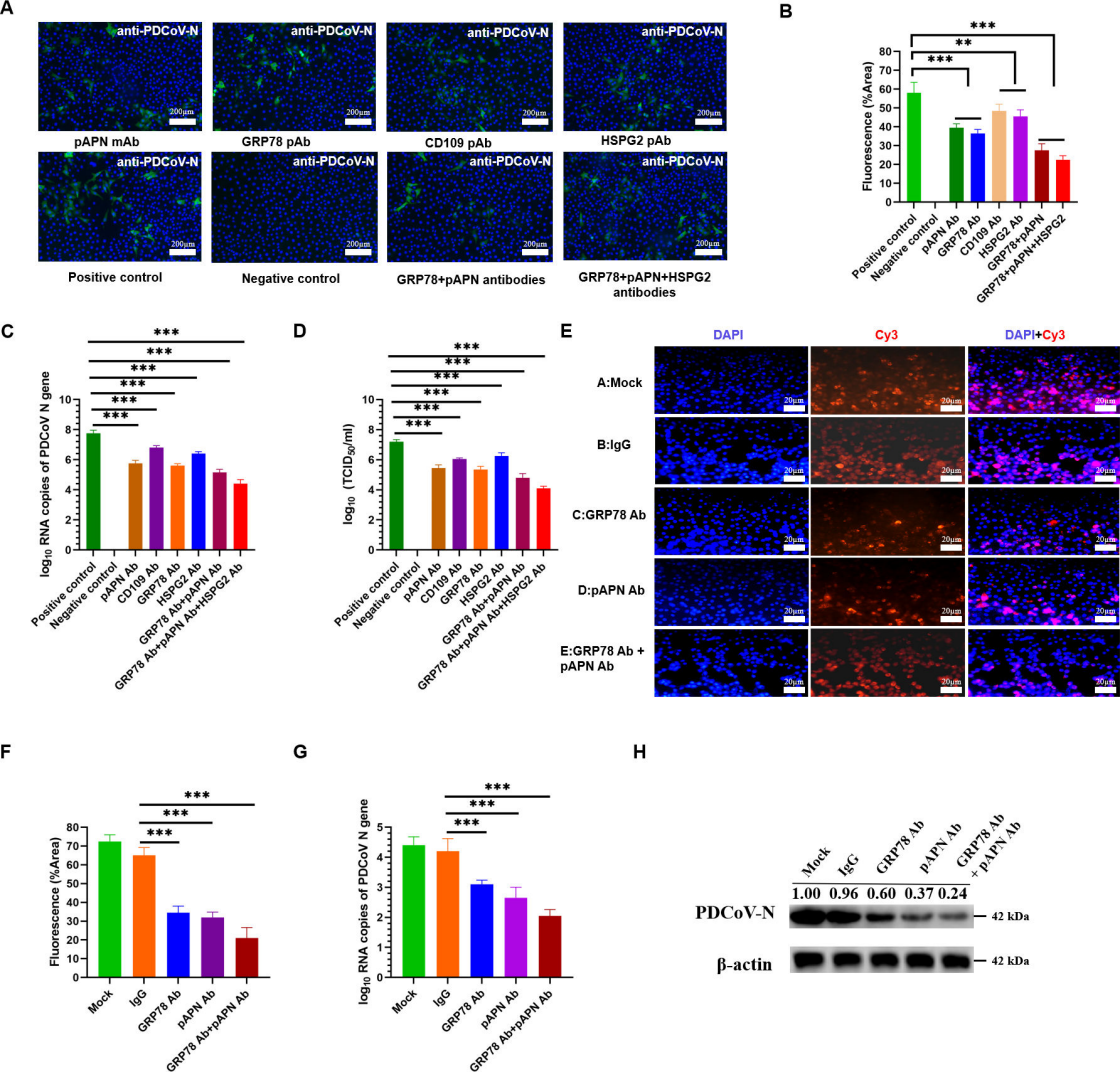
Blocking GRP78 rendered PDCoV cell attachment and entry. (**A**) Immunofluorescence detection of the PDCoV N protein in IPEC-J2 cells infected with the virus after incubation with antibodies specific to pAPN, GRP78, CD109, and HSPG2. Antibody combinations are denoted with a plus sign. Cells incubated with nonspecific mouse IgG were used as a positive control and uninfected cells were used as a negative control. Magnification: 100×. (**B**) Fluorescence intensity measurements from the experiments shown in panel A using ImageJ software. (**C** and **D**) PDCoV N gene copy number and PDCoV titers in antibody-blocked IPEC-J2 cells, as measured by TCID₅₀ and real-time RT-PCR, respectively. (**E**) The amounts of PDCoV particles in IPEC-J2 cells surface blocked with anti-GRP78, anti-pAPN, or both antibodies were detected by RNAscope *in situ* hybridization. The mouse IgG was set as a negative control. Red fluorescence indicates the viral particle signal. Magnification: 100×. (**F**) Statistical analysis of fluorescence intensity measurements from multiple RNAscope images. (**G**) A real-time RT-PCR measurement of the PDCoV N gene copy number reflects the number of viral particles attached to the surface of antibody-treated IPEC-J2 cells. (**H**) A similar analysis was performed using Western blotting with an anti-PDCoV-N antibody. The intensity of protein bands was measured using ImageJ software. Data are presented as means ± SD and are representative of at least two independent experiments. *P < 0.05, **P < 0.01, ***P < 0.001 (unpaired Student’s *t*-test).

To elucidate the roles of pAPN, GRP78, CD109, and HSPG2 in PDCoV cell attachment, special antibody blocking assays were applied. IPEC-J2 cells were pre-incubated with antibodies against pAPN, GRP78, CD109, or HSPG2 before infection with PDCoV. Viral infection was subsequently assessed by IFA, quantitative real-time RT-PCR, and TCID_50_ assays. The IFA results showed that incubating the cells with pAPN and GRP78 antibodies significantly inhibited PDCoV infection compared to incubation with isotype IgG. Furthermore, combination treatments produced an additive inhibitory effect (Fig. 2A and B). Similar results were obtained using quantitative real-time RT-PCR (Fig. 2C) and TCID_50_ assays (Fig. 2D). Collectively, these results suggested that GRP78, similar to the documented pAPN, plays important roles in viral attachment and entry.

Subsequently, we employed *in situ* hybridization technique RNAscope to quantify the number of viruses adsorbed on the cell membrane, thereby further elucidating the role of GRP78 in the adsorption of PDCoV. After pre-incubating the cells with antibodies against pAPN and/or GRP78 at 37℃ for 1 h, we incubated them with PDCoV at 4℃ for 2 h, which is sufficient time for the virus to attach to the cells. The cells were then washed, fixed, and subjected to RNAscope analysis. The analysis revealed significantly reduced PDCoV attachment (represented by red fluorescence) in cells pretreated with antibodies, particularly those pretreated with the pAPN and GRP78 antibody combination (Fig. 2E and F). Similar results were obtained by quantitative real-time RT-PCR and Western blot analysis of membrane-associated PDCoV (Fig. 2G and H), supporting the conclusion that GRP78 is essential for PDCoV cell attachment.

**Fig 3:**
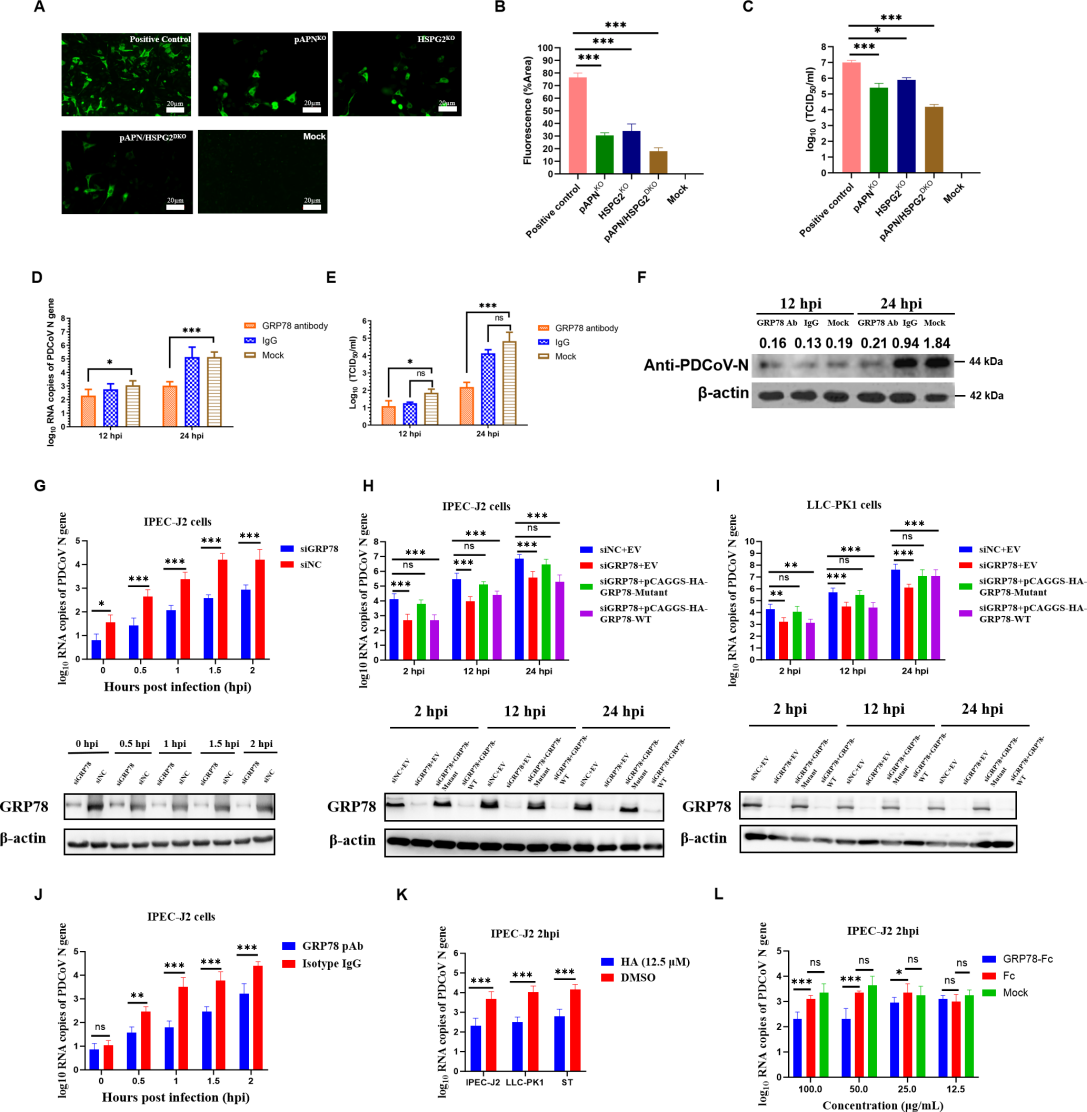
Antibody blocking of GRP78 on the surface of pAPN/HSPG2 double knockout cells inhibits PDCoV infection. (**A**) PDCoV infection in IPEC-J2 pAPN^KO^, IPEC-J2 HSPG2^KO^, and IPEC-J2 (pAPN/HSPG2)^DKO^ cells was examined by IFA using an anti-PDCoV N antibody. Magnification: 100×. (**B**) Fluorescence intensity measurements from multiple images shown in panel A, as determined using ImageJ software. (**C**) PDCoV titers in IPEC-J2 pAPN^KO^, IPEC-J2 HSPG2^KO^, and IPEC-J2 (pAPN/HSPG2)^DKO^ cells, as measured by the TCID_50_ assay. (**D**, **E**, and **F**) The effect of GRP78 blocking with a specific antibody on PDCoV infection of the double knockout cells. Shown are the results of the TCID₅₀ assay, quantitative real-time RT-PCR, and Western blotting, respectively. (**G**) Following GRP78-specific RNA interference, IPEC-J2 cells were infected with PDCoV at an MOI of 1 and harvested at 0, 0.5, 1, 1.5, and 2 hpi. Viral RNA was extracted, and the copy number of the viral N gene was determined by RT-qPCR. The knockdown levels of GRP78 were determined by WB. (**H** and **I**) GRP78 protein knockdown rescue experiment. IPEC-J2 (**H**) and LLC-PK1 (**I**) cells were transfected with a GRP78 overexpression plasmid that is resistant to GRP78-targeting RNA interference. At 48 h post-transfection, cells were inoculated with PDCoV at an MOI of 1 and harvested at 2, 12, and 24 hpi. After viral RNA was extracted, the copy number of the viral N gene was determined by RT-qPCR. The expression levels of GRP78 were determined by WB. (**J**) After blockade with a GRP78-specific antibody, cells were inoculated with PDCoV at an MOI of 1 and harvested at 0, 0.5, 1, 1.5, and 2 hpi. After viral RNA was extracted, the copy number of the viral N gene was determined by RT-qPCR. (**K**) The copy number of the viral N gene was determined by RT-qPCR in different cell lines infected with PDCoV at 2 hpi, which were treated with the GRP78-specific inhibitor HA15. (**L**) IPEC-J2 cells were infected with PDCoV which was pre-incubated with various concentrations of GRP78-Fc protein and harvested at 2 hpi. The copy number of the viral N gene was determined by RT-qPCR. Data are presented as means ± SD and are representative of at least two independent experiments. ns represents no significance, *P < 0.05, **P < 0.01, ***P < 0.001 (unpaired Student’s *t*-test).

Next, to obtain more definitive effects of GRP78 on PDCoV infection, we employed the CRISPR/Cas9 system to generate GRP78 knockout IPEC-J2 cells (GRP78^KO^). Unfortunately, we failed to obtain GRP78^KO^ monoclonal cell lines, as all cells died following GRP78 knockout. This is consistent with the fact that GRP78 is essential for cell viability (46, 47). Therefore, we employed an alternative approach to study the impact of GRP78 depletion in knockout cells. we knocked out other factors that aid in viral adsorption and entry and then blocked with GRP78-specific antibodies to further observe the extent of reduction in viral titer. As a result, the IPEC-J2 pAPN^KO^, IPEC-J2 HSPG2^KO^, and IPEC-J2 (pAPN/HSPG2)^DKO^ cells were achieved and confirmed by Western blotting (Fig. S1C) and nucleotide sequencing. We infected the knockout cells with PDCoV and assessed infection using IFA and TCID_50_ assays. Both approaches revealed a significant inhibition of PDCoV infection in all knockout cells compared to wild-type IPEC-J2 cells (Fig. 3A through C). These results confirmed the importance of pAPN and HSPG2 for PDCoV infection. After that, we blocked GRP78 on the surface of IPEC-J2 (pAPN/HSPG2)^DKO^ cells, and the titers of PDCoV were detected by real-time RT-PCR (Fig. 3D), the TCID_50_ assay (Fig. 3E), and Western blotting (Fig. 3F). The results revealed a significant inhibition of PDCoV infection in IPEC-J2 (pAPN/HSPG2)^DKO^ cells pretreated with the anti-GRP78 antibody compared to those pretreated with nonspecific IgG or untreated controls (Fig. 3D through F). These findings provide further evidence that GRP78 serves as a critical host factor for PDCoV.

To determine whether GRP78 is involved in the internalization step of PDCoV entry, we performed a series of internalization assays using the classical 4°C binding followed by 37°C internalization protocol. PDCoV was allowed to bind to IPEC-J2 cells at 4°C for 1 h, during which the virus can attach to the cell surface but cannot internalize. After removing unbound virus, cells were shifted to 37°C for various time points (0, 0.5, 1, 1.5, and 2 h) to permit internalization. Cells were then treated with proteinase K to eliminate surface-bound virus, and internalized viral RNA levels were quantified using RT-qPCR. As shown in Fig. 3G, GRP78 knockdown significantly reduced the levels of internalized viral RNA at 1 h and 2 h post-internalization compared with the control group, directly demonstrating a critical role for GRP78 in the viral internalization step. To further exclude the possibility of off-target effects from GRP78 siRNA and to establish the specific role of GRP78 in PDCoV infection, we constructed an siRNA-resistant GRP78 overexpression plasmid. Using site-directed mutagenesis, we introduced 3–4 synonymous mutations into the siRNA recognition region of the GRP78 coding sequence (GAA→GAG, CTC→CTA, TTT→TTC), ensuring that the mutated mRNA was no longer complementary to the siRNA while maintaining an amino acid sequence identical to that of wild-type GRP78. The mutated sequence was then cloned into the pCAGGS-HA vector to generate the GRP78-rescue plasmid, and its expression was confirmed by Western blotting (Fig. 3H and I). We next performed rescue experiments in IPEC-J2 and LLC-PK1 cells. Cells were divided into four groups: (i) control siRNA + empty vector (control group); (ii) GRP78 siRNA + empty vector (knockdown group); (iii) GRP78 siRNA + GRP78-rescue plasmid (rescue group); and (iv) GRP78 siRNA + wild-type GRP78 overexpression plasmid (wild-type overexpression group). At 48 h post-transfection, cells were infected with PDCoV, and viral N protein expression was assessed. Western blotting analysis confirmed that GRP78 protein levels were markedly reduced in the knockdown group compared with the control group. Importantly, co-transfection of the GRP78-rescue plasmid effectively restored GRP78 expression (Fig. 3H and I). As shown in both cell lines, PDCoV infection levels were significantly reduced in the knockdown group compared with the control group. Notably, the rescue group exhibited a marked restoration of viral infectivity, with N protein expression levels comparable to those of the control group and significantly higher than those of the knockdown group. In contrast, co-transfection of the wild-type GRP78 overexpression plasmid failed to rescue infectivity, showing no significant difference from the knockdown group, thereby confirming that the wild-type GRP78 was effectively knocked down by the siRNA (Fig. 3H and I). Collectively, these results demonstrate that the reduction in PDCoV infection is specifically attributed to GRP78 knockdown, rather than off-target effects of the siRNA. Furthermore, we investigated whether GRP78 knockdown affects the protein expression level and cellular distribution of pAPN, thereby leading to reduced viral internalization capacity. The results showed that GRP78 knockdown did not affect pAPN expression (Fig. S1D) or its cellular distribution (Fig. S1E). This indicates that the reduced viral internalization efficiency caused by GRP78 knockdown is independent of pAPN expression and distribution.

Consistent with this, pretreatment of IPEC-J2 cells with an anti-GRP78 antibody also markedly decreased internalized viral RNA levels at 1 h and 2 h (Fig. 3J), further supporting the functional involvement of GRP78 in viral internalization. We next evaluated the effect of HA15, a specific small-molecule inhibitor of the GRP78 protein (48), on viral internalization in different cell lines. Cytotoxicity assay showed that 12.5 μM HA15 had no significant cytotoxicity on all three cell lines (Fig. S2A). At 2 h post-infection, cells were treated with proteinase K to remove surface-bound virus, and intracellular viral RNA was extracted. RT-qPCR quantification of PDCoV N gene copy numbers revealed that HA15 treatment significantly inhibited viral internalization (Fig. 3K). Similarly, pre-treating viral particles with soluble GRP78 protein also significantly reduced viral infectivity (Fig. 3L). Collectively, these results establish that GRP78 is critically required for the internalization phase of PDCoV entry.

### GRP78 protein interacts with PDCoV S1 via the SBD domain

**Fig 4:**
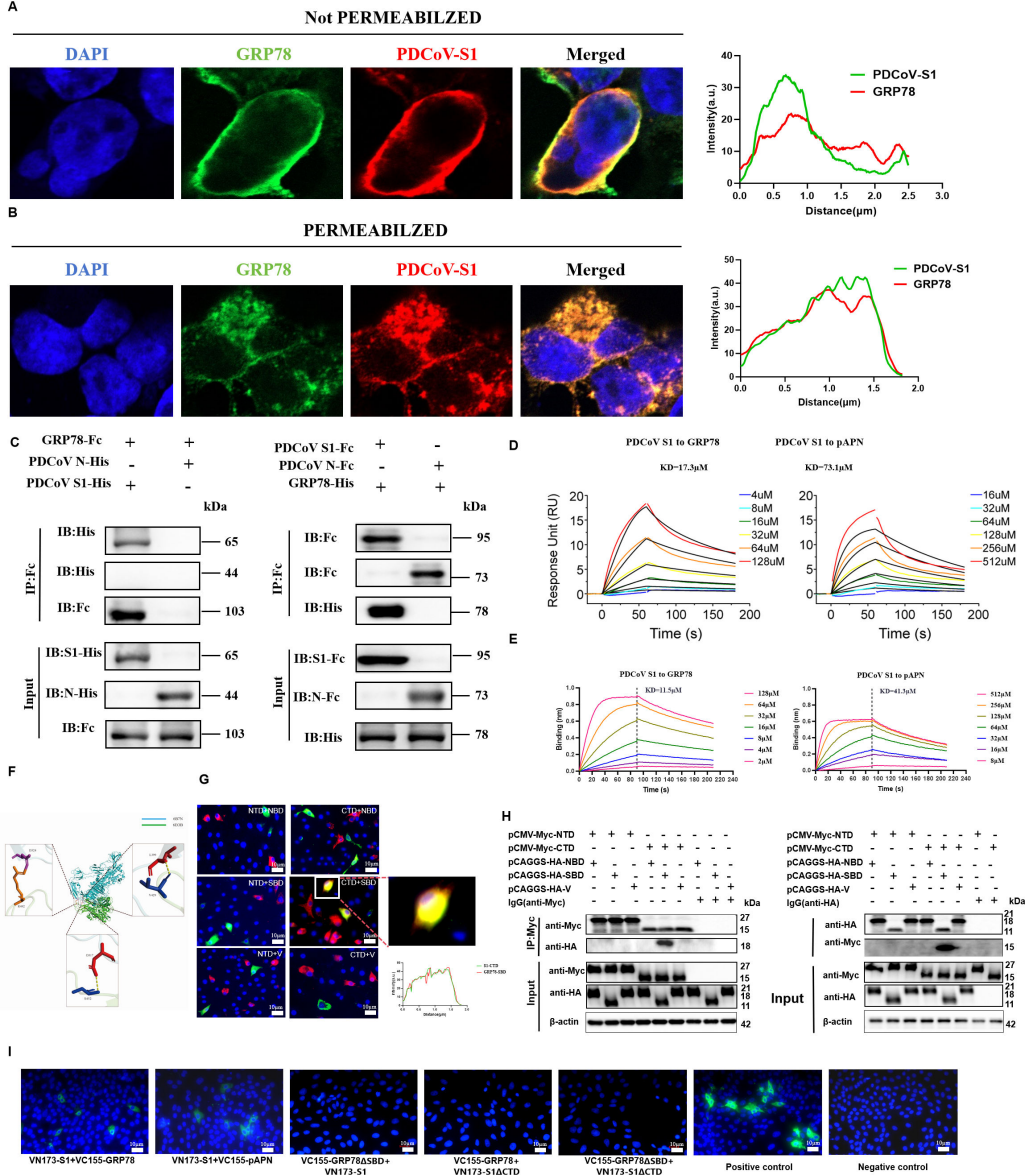
GRP78 protein interacts with PDCoV S1 protein via the SBD domain. (**A** and **B**) Co-localization of PDCoV S1 with endogenous GRP78 in non-permeabilized (**A**) and permeabilized (**B**) virus-infected IPEC-J2 cells. Immunofluorescence microscopy images were obtained using antibodies against S1 and GRP78. Magnification: 1,000×. Protein colocalization was analyzed by line scanning analysis. (**C**) The interaction between GRP78 and PDCoV S1 protein was determined by Co-IP assay. (**D** and **E**) Binding affinity of PDCoV S1 to GRP78 or pAPN was measured by SPR and BLI, respectively. (**F**) PyMOL software predicted interaction sites between the substrate-binding domain (SBD) of GRP78 and the C-terminal domain (CTD) of PDCoV S1. (**G**) Plasmids expressing the HA-tagged NBD, SBD, and V domains of GRP78, as well as the Myc-tagged NTD and CTD domains of PDCoV S1 were co-transfected into BHK-21 cells in pairwise comparison, which were subsequently analyzed by immunofluorescence microscopy to detect protein co-localization. Red denotes HA-tagged proteins, and green denotes Myc-tagged proteins. Yellow indicates protein co-localization, suggesting a protein-protein interaction. Protein-protein interactions were also assessed by line scan analysis. Magnification 200×. (**H** and **I**) Validation of the interaction between the SBD domain of GRP78 and the CTD domain of PDCoV S1 using Co-IP assays and BiFC assays, respectively. In BiFC assays, the positive signal was indicated by green fluorescence. Strongly interacting b-Jun and b-Fos were used as a positive control. The lack of interaction between b-Jun and the b-Fos mutant deltaZIP served as a negative control. Magnification: 200×.

After demonstrating that GRP78 is involved in PDCoV cell entry, we sought to examine the interaction between GRP78 and the PDCoV S1 protein in greater detail. First, we investigated whether these two proteins co-localize within infected IPEC-J2 cells. To this end, we analyzed permeabilized and non-permeabilized PDCoV-infected cells using double immunofluorescence microscopy with anti-GRP78 and anti-PDCoV S1 antibodies. We validated the quality of our anti-PDCoV S1 antibody using Western blotting (Fig. S2B) and IFA (Fig. S2C). Using high-resolution confocal imaging, we found that GRP78 and the PDCoV S1 protein exhibited significant colocalization at the surface and the plasma membrane region of infected cells. Line scan analysis further confirmed the high coincidence of fluorescence intensity peaks at the surface and the plasma membrane (Fig. 4A and B). In addition, we measured the GRP78 levels on the cell surface and in the cytoplasm by cell surface protein extraction and Western blot assays. By densitometric quantification, we calculated that cell surface GRP78 accounted for approximately 4.8% ± 0.7% (IPEC-J2 cells), 5.2% ± 0.9% (LLC-PK1 cells), and 11.4% ± 0.4% (ST cells) of total cellular GRP78 protein (mean ± SD, *n* = 3 independent experiments) (Fig. S2D).

Another lines of evidence supporting the interaction between GRP78 and PDCoV S1 were obtained using a Co-IP assay and a protein affinity assay with purified proteins. Specifically, purified GRP78-Fc was immobilized on Protein A/G magnetic beads and incubated with either purified His-tagged PDCoV-S1 or PDCoV-N (as a negative control). Western blot analysis of the bead eluates revealed that PDCoV-S1, but not PDCoV-N, could bind to GRP78. In a complementary approach, purified S1-Fc protein (or the control N-Fc protein) was immobilized on Protein A/G magnetic beads and incubated with GRP78-His. The results showed that S1-Fc beads efficiently pulled down GRP78-His protein. In contrast, the control N-Fc beads failed to pull down GRP78-His, thereby ruling out non-specific binding mediated by beads (Fig. 4C). Meanwhile, surface plasmon resonance (SPR) results showed that the affinity (KD value) between GRP78 and the S1 protein was 17.3 µM, whereas the affinity between pAPN and S1 was 73.1 µM (Fig. 4D). Bio-layer interferometry (BLI) results revealed a KD value of 11.5 μM for S1-GRP78 binding, which is consistent with the SPR result (17.3 μM) within an acceptable experimental error range. The KD value for S1-pAPN binding was 41.3 μM, also generally consistent with the SPR result (73.1 μM) (Fig. 4E). Both assays confirmed that the affinity of S1 for GRP78 is stronger than that for pAPN. Collectively, these findings provide additional support for a specific interaction between GRP78 and PDCoV S1.

Our next goal was to map the functional domains involved in the interaction between the PDCoV S1 protein and GRP78. The GRP78 protein comprises three functional domains: the nucleotide-binding domain (NBD), the substrate-binding domain (SBD), and the variable domain (49). Similarly, the PDCoV S1 protein is composed of two domains: the N-terminal domain (NTD) and the C-terminal domain (CTD). *In silico* predictions suggested the existence of potential binding sites within the SBD domain of GRP78 (residues S452, R492, and V429) and the CTD domain of S1 (residues D317, D324, and L399) (Fig. 4F). To validate these predictions, we expressed each GRP78 and S1 domain independently in BHK-21 cells. The GRP78 and S1 domains were tagged with HA and Myc epitopes, respectively. We validated protein expression from these plasmids in BHK-21 cells (Fig. S2E). Immunofluorescence microscopy revealed that the S1-CTD and GRP78-SBD proteins co-localized in transfected BHK-21 cells. In contrast, no co-localization was observed for the other domains. Line scan analysis also revealed a large number of overlapping peaks between the SBD domain and the CTD domain of GRP78 (Fig. 4G). These results suggested that the interaction between S1 and GRP78 occurs via the CTD and SBD domains. Further support for this possibility was obtained through Co-IP assays, where the S1-CTD/GRP78-SBD complex was successfully immunoprecipitated using both anti-HA and anti-Myc antibodies (Fig. 4H). Based on these results, we concluded that the binding of the PDCoV spike protein to the GRP78 through the corresponding CTD and SBD domains.

To validate the specificity and domain dependence of the interaction between PDCoV S1 and GRP78, we performed BiFC assays. The expression of all targeted fusion proteins was confirmed by immunofluorescence microscopy using anti-HA and anti-FLAG antibodies (Fig. S2F). We used the strongly interacting b-Jun and b-Fos transcription factors as a positive control and the b-Fos mutant lacking the bZIP domain as a negative control. As a result, it showed that successful assembly of the fluorescent reporter protein fragments tethered to pAPN and PDCoV S1, as well as to GRP78 and PDCoV S1, within BHK-21 cells was observed. While, co-expression of wild-type S1 with the GRP78 mutant lacking the SBD domain, or co-expression of the S1 mutant lacking the CTD domain with wild-type GRP78, resulted in no detectable BiFC fluorescence signal (Fig. 4I). These results confirm that S1-CTD and GRP78-SBD are necessary and sufficient domains for their interaction.

### GRP78 facilitates PDCoV internalization in a species- and tissue-independent manner

Validating GRP78 function in primary porcine cells and across a broader range of cell lines is critically important for establishing its universal role as a key entry factor for PDCoV. Therefore, we acquired porcine primary cells (PIG-iCell-d007) and selected the following cell lines across different species and tissue origins: porcine cell lines IPEC-J2 (intestinal epithelium), LLC-PK1 (kidney epithelium), PK-15 (kidney epithelium), and ST (testicular fibroblasts); human cell lines Huh-7 (hepatoma), Caco-2 (colonic adenocarcinoma), and HEK293T (embryonic kidney); and other species cell lines VeroE6 (African green monkey kidney) for functional validation of GRP78.

**Fig 5:**
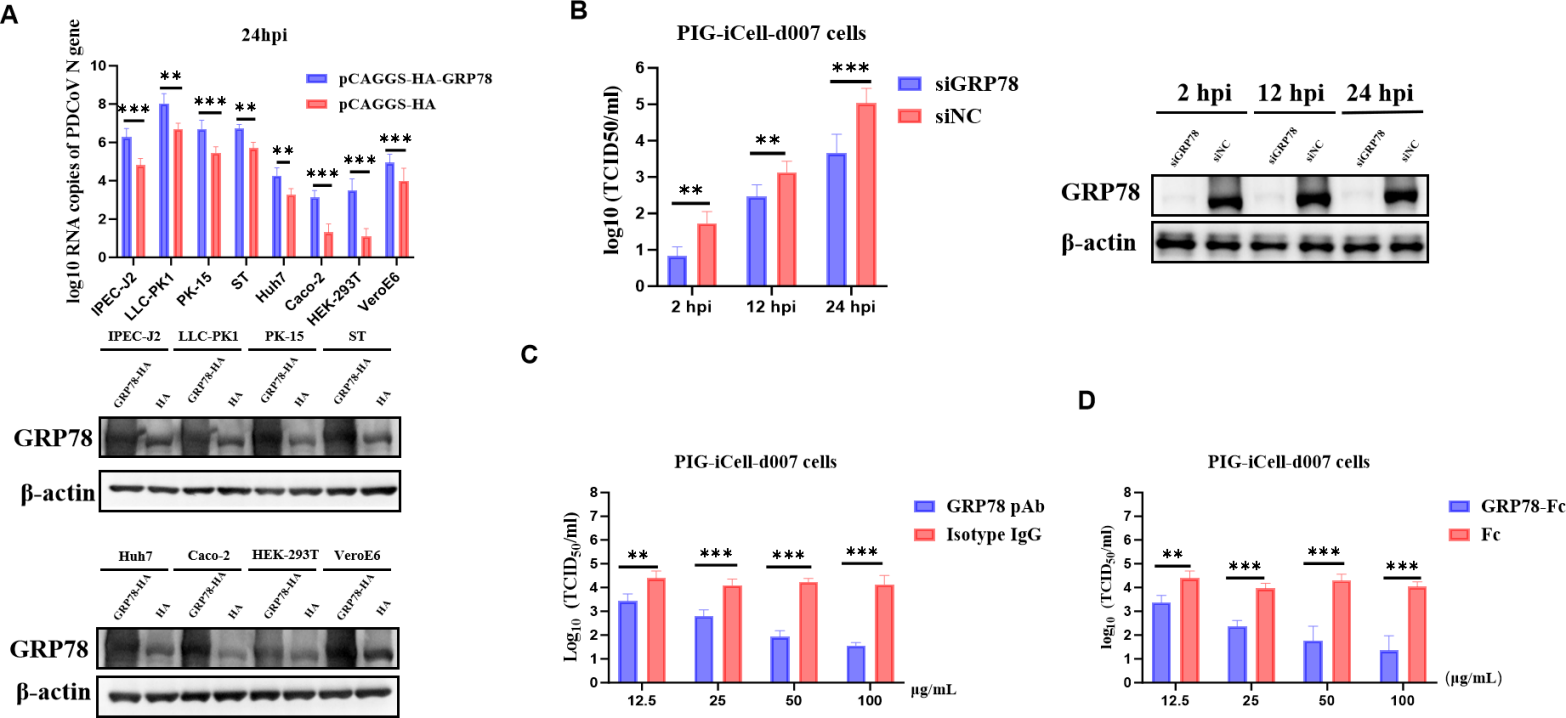
GRP78 mediates PDCoV infection in multiple cell lines and primary porcine intestinal epithelial cells. (**A**) Multiple cell lines were infected with PDCoV after overexpressing porcine GRP78 protein and harvested at 2 hpi. The copy number of the viral N gene was determined by RT-qPCR. GRP78 protein expression levels were measured by WB. (**B**) RNA interference assays were carried out in primary porcine intestinal epithelial cells with GRP78 knock-down by RNA interference assay were infected with PDCoV at an MOI of 1. Cells were harvested at 2 hpi, 12 hpi, and 24 hpi, respectively. The copy number of the viral N gene was determined by RT-qPCR. (**C**) Primary porcine intestinal epithelial cells were blocked with various concentrations of a GRP78-specific antibody and then infected with PDCoV. Isotype IgG-treated group were set as a isotype control. At 2 hpi, cells were harvested and viral RNA was extracted. The copy number of the viral N gene was determined by RT-qPCR. (**D**) Primary porcine intestinal epithelial cells were inoculated with PDCoV pre-incubated with GRP78 protein. At 2 hpi, the cells were harvested, and the copy number of the viral N gene was determined by RT-qPCR. Data are presented as means ± SD and are representative of at least two independent experiments. **P < 0.01, ***P < 0.001 (unpaired Studentʼs *t*-test).

In different species cell lines, GRP78 overexpression plasmids (or empty vector controls) were transiently transfected. At 48 h post-transfection, cells were inoculated with PDCoV for 2 h to assess viral internalization efficiency. Our results showed that in porcine cell lines (IPEC-J2, LLC-PK1, PK-15, ST), GRP78 overexpression increased PDCoV internalization efficiency by 2.1- to 2.8-fold. While, in human cell lines (Huh-7, Caco-2, HEK293T) and VeroE6 cells, GRP78 overexpression also increased PDCoV internalization efficiency by 1.5- to 2.2-fold and 1.3- to 1.6-fold, respectively (Fig. 5A), indicating that the entry-promoting function of GRP78 is not limited to porcine cells.

In primary porcine intestinal epithelial cells (PIG-iCell-d007), siRNA knockdown, specific antibody blocking, and soluble protein blocking assays were performed. In siRNA knockdown assays, cells were transfected with GRP78 siRNA or control siRNA using an optimized lipofection protocol. At 48 h post-transfection, knockdown efficiency was verified by Western blotting. Subsequently, cells were inoculated with PDCoV to assess viral internalization efficiency (viral titers at 2 h), replication levels (viral titers at 12 h), and release levels (viral titers at 24 h). The results demonstrated that GRP78 siRNA achieved a knockdown efficiency of more than 90% in the primary cells and the internalization efficiency and viral titers were significantly reduced, which is consistent with the results observed in IPEC-J2 cells (Fig. 5B). In specific antibody blocking assays, primary cells were pre-treated with various concentrations of anti-GRP78 antibody (recognizing the extracellular domain) or isotype control IgG for 1 h, followed by PDCoV inoculation. Cells were harvested at 2 hpi to assess viral attachment and internalization efficiency. As a consequence, the anti-GRP78 antibody significantly inhibited PDCoV attachment and internalization in primary cells in a dose-dependent manner compared with the isotype control IgG group (Fig. 5C). In soluble protein blocking assays, PDCoV was pre-incubated with various concentrations of soluble GRP78-Fc protein and Fc protein for 1 h at 37°C. The mixture was then added to primary cells, and cells were harvested at 2 hpi to assess viral attachment and internalization efficiency. Soluble GRP78-Fc significantly inhibited PDCoV attachment and internalization in primary cells in a dose-dependent manner compared with the Fc-treated group (Fig. 5D). These results indicate that the GRP78 protein plays the same role in promoting PDCoV internalization in primary cells.

### GRP78 mediates PDCoV cell entry primarily through clathrin-mediated endocytosis and in a pAPN-independent pathway

**Fig 6:**
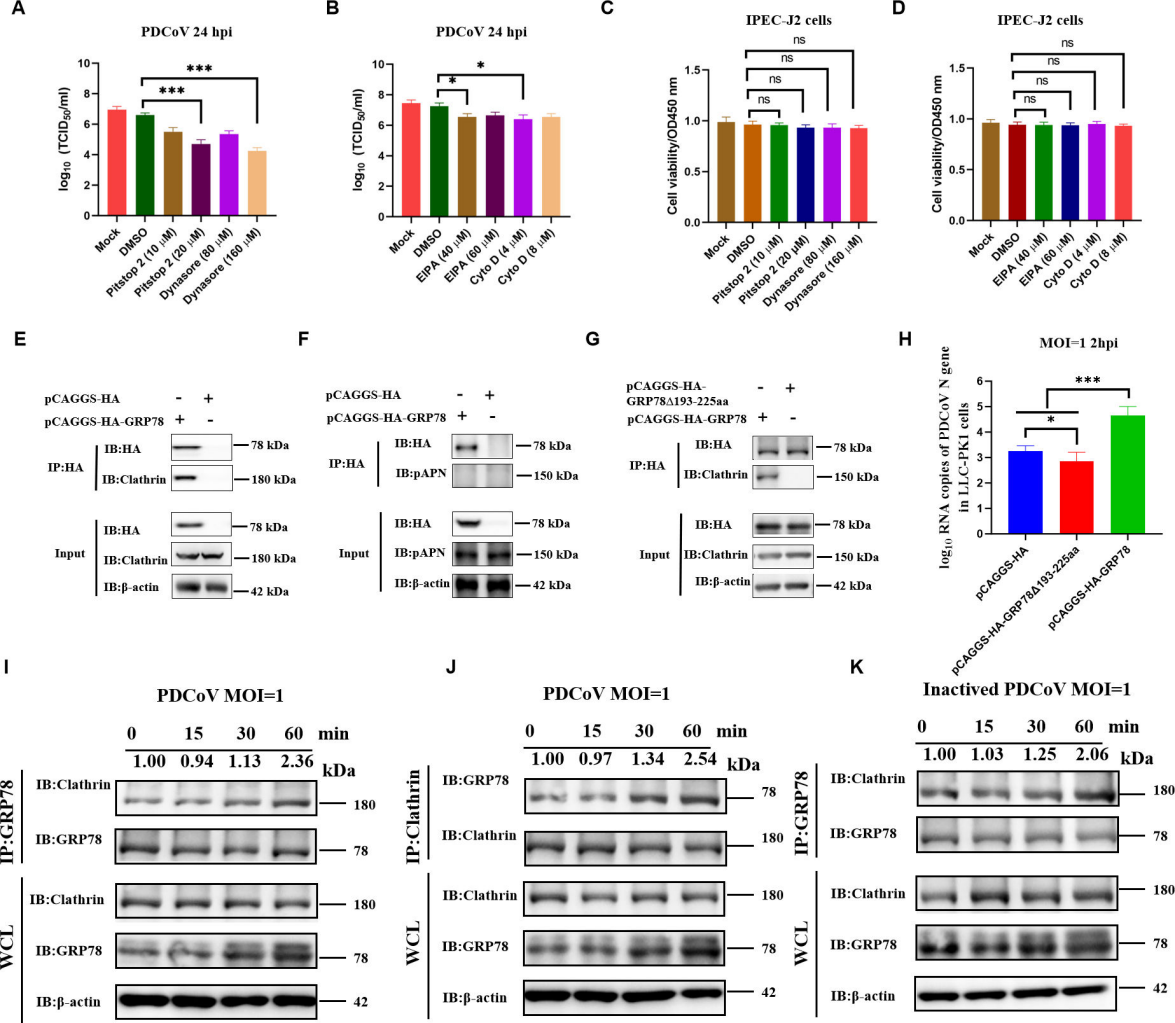
GRP78 mediates PDCoV cell entry primarily through the clathrin-mediated endocytosis pathway. (**A**) IPEC-J2 cells were incubated with the clathrin inhibitors Pitstop 2 (10 µM and 20 µM) or Dynasore (80 µM and 160 µM) and then inoculated with PDCoV. PDCoV titers were subsequently determined using the TCID_50_ assay. DMSO was used as a negative control. (**B**) IPEC-J2 cells were incubated with the macropinocytosis inhibitors EIPA (40 µM and 60 µM) or Cytochalasin D (Cyto D; 4 µM and 8 µM), inoculated with PDCoV, and viral titers were subsequently measured by the TCID₅₀ assay. (**C** and **D**) Cell viability was measured using the CCK-8 kit after treatment with the clathrin and macropinocytosis inhibitors, respectively. (**E** and **F**) The interaction between GRP78 and clathrin or pAPN was measured by Co-IP assay. (**G**) The interaction of the GRP78 mutant with clathrin was assessed by WB. (**H**) LLC-PK1 cells were transfected with GRP78 mutant plasmid or GRP78 wild-type plasmid and then infected with PDCoV. The copy number of the viral N gene was determined by RT-qPCR. (**I** and **J**) IPEC-J2 cells were infected with PDCoV and harvested at 0, 15, 30, and 60 min post-infection. After lysis, Co-IP assays were performed using GRP78 antibody (**I**) or clathrin antibody (**J**) to determine the changes in the interaction intensity between GRP78 and clathrin. (**K**) IPEC-J2 cells were infected with inactivated PDCoV and harvested at 0, 15, 30, and 60 min post-infection. After lysis, Co-IP assays were performed using GRP78 antibody to determine the changes in the interaction intensity between GRP78 and clathrin. Data are presented as means ± SD and are representative of at least two independent experiments. ns represents no significance, *P < 0.05, ***P < 0.001 (unpaired Studentʼs *t*-test).

After binding to cell surface, the virus needs a specific cell entry pathway for internalization. However, the internalization mechanism by which PDCoV enters cells through binding to GRP78 remains unclear. Therefore, our next aim was to determine whether PDCoV uses clathrin-mediated endocytosis and/or macropinocytosis for cell entry. To answer this question, first, we employed inhibitors that specifically target these pathways. The results showed that treatment with Pitstop 2 or Dynasore, which inhibit clathrin-mediated endocytosis, significantly reduced PDCoV titers, as measured by the TCID_50_ assay (Fig. 6A). Treatment with EIPA and cytochalasin D (Cyto D), which inhibit macropinocytosis, caused only a slight decrease in PDCoV titers (Fig. 6B). These results suggested that PDCoV primarily enters host cells via clathrin-mediated endocytosis. Furthermore, to exclude the interference of drug off-target effects, based on previous studies (50, 51), we selected PEDV, which is known to enter cells through internalization via this pathway, as a pathway-specific positive control. Under the same experimental conditions, Pitstop 2 and Dynasore significantly inhibited PEDV internalization (Fig. S3A and B). This result demonstrates that, at the inhibitor concentrations used in this study, their effects are pathway-specific, ruling out major concerns regarding non-specific off-target effects. These results suggested that macropinocytosis plays a secondary role in PDCoV entry. Cytotoxicity measurements using the CCK-8 kit confirmed that the drug concentrations used had no effect on cell viability (Fig. 6C and D; Fig. S3C and D). Collectively, these results revealed that clathrin-mediated endocytosis is the primary pathway for PDCoV cell entry.

To investigate the functional relationship between GRP78 and the clathrin-mediated endocytic machinery, we examined whether GRP78 interacts with clathrin. Co-immunoprecipitation (Co-IP) assays revealed that GRP78 could interact with clathrin and does not interact with pAPN (Fig. 6E and F). We then examined whether GRP78 knockdown affects the expression and subcellular distribution of pAPN. The results showed that both the expression and cellular distribution of pAPN were unaffected by GRP78 levels, indicating that GRP78 and pAPN function independently (Fig. S3E and F). To further determine whether the GRP78-clathrin interaction is functionally relevant during viral entry, we constructed a GRP78 mutant GRP78∆193-225aa to disrupt the coupling between GRP78 and clathrin, thereby validating the specificity of the pathway. As shown in Fig. 6G, the GRP78∆193-225aa could not interact with clathrin efficiently. Subsequently, LLC-PK1 cells were separately transfected with this mutant and wild-type GRP78 plasmids. At 48 h post-transfection, the cells were infected with PDCoV. Cells were harvested at 2 hpi to determine PDCoV viral titer. The results showed that the viral titer in the mutant-transfected group was significantly lower than that in the wild-type GRP78 plasmid-transfected group (Fig. 6H). Moreover, we performed time-course Co-IP experiments comparing uninfected and PDCoV-infected cells. The results demonstrated that following PDCoV infection, the GRP78-clathrin interaction was significantly enhanced, peaking at 60 min post-infection with approximately a 2.3-fold increase compared to 0 min (*P* < 0.01) (Fig. 6I). The reverse Co-IP assays showed the similar result (Fig. 6J). Notably, UV-inactivated virus also induced an enhancement of the GRP78-clathrin interaction (approximately 2.0-fold), indicating that the induction event occurs at the viral binding stage and does not depend on viral replication (Fig. 6K). These results support a model in which viral binding triggers increased recruitment of or binding affinity between GRP78 and clathrin, thereby coupling the virus-receptor complex to the endocytic machinery to facilitate PDCoV internalization.

### GRP78 protein promotes the infection of other coronaviruses

**Fig 7:**
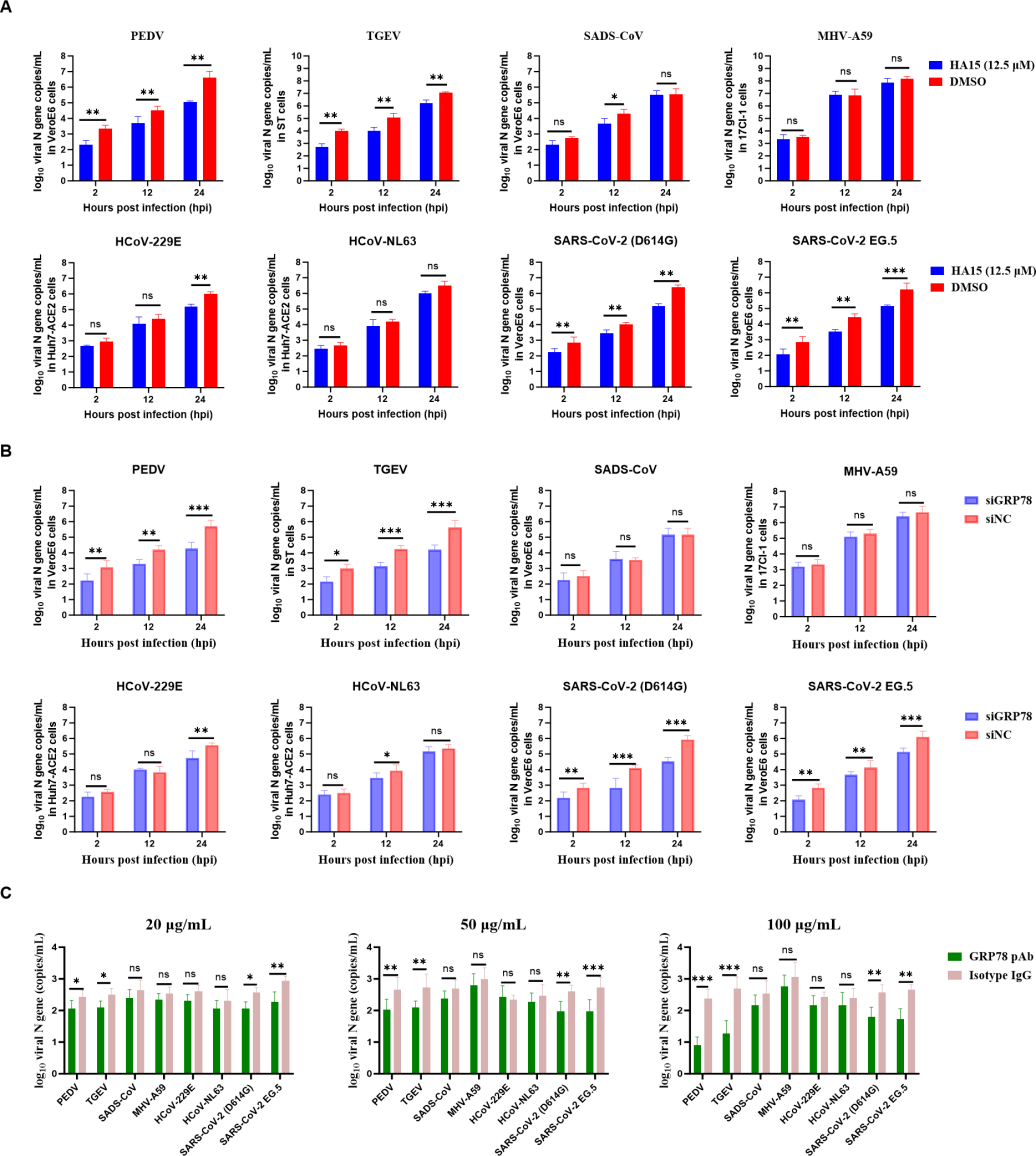
GRP78 protein promotes the infection of other coronaviruses. (**A** and **B**) After HA15 treated or GRP78 expression konck down, the cells were collected at 2 hpi, 12 hpi, and 24 hpi, and viral titers were determined by RT-qPCR, respectively. (**C**) Cells were blocked with various concentrations of GRP78 special antibody, followed by inoculation with different coronaviruses for 2 h. The cells were then harvested, and viral titers were measured by RT-qPCR, with isotype IgG antibody used as a negative control. Data are presented as means ± SD and are representative of at least two independent experiments. ns represents no significance, *P < 0.05, **P < 0.01, ***P < 0.001 (unpaired Student’s *t*-test).

To determine whether other coronaviruses, including porcine epidemic diarrhea virus (PEDV), swine acute diarrhea syndrome coronavirus (SADS-CoV), transmissible gastroenteritis virus (TGEV), mouse hepatitis virus (MHV-A59), human coronavirus 229E (HCoV-229E), human coronavirus NL63 (HCoV-NL63), and SARS-CoV-2 also can utilize GRP78 for cell entry, we treated host cells with HA15 and then infected the cells with the virus at an MOI of 1 for 1 h absorption. After that, the media were replaced with HA15, and the cells and supernatants were collected at 2 hpi, 12 hpi, and 24 hpi for the detection of viral copy number using absolute quantitative real-time PCR. The results showed that PEDV, TGEV, and SARS-CoV-2 were sensitive to GRP78 intervention, with entry efficiency reduced by 50%–70%. In contrast, SADS-CoV, MHV, HCoV-229E, and HCoV-NL63 were relatively insensitive (reduction <20%, not statistically significant). These findings indicate that while GRP78 promotes the entry of multiple coronaviruses, the extent of dependency varies among different viruses. Thus, GRP78 may serve as a broad-spectrum anti-coronavirus target although its applicability should consider virus-specific differences (Fig. 7A).

Subsequently, to provide robust evidence for the role of GRP78 in the entry of multiple coronaviruses, two orthogonal strategies, siRNA knockdown and antibody blockade, were performed. The GRP78 interfering RNA (siGRP78) was transfected into virus-susceptible cell lines, and the expression levels of GRP78 protein were detected at 48 h post-transfection. Cells were then inoculated with virus at an MOI of 1 and collected at 2, 12, and 24 hpi for viral titer determination. The results showed that transfection with siGRP78 effectively reduced GRP78 protein expression (Fig. S3E). Under these conditions, the internalization titers of PEDV, TGEV, and SARS-CoV-2 were significantly reduced, whereas those of SADS-CoV, MHV, HCoV-229E, and HCoV-NL63 remained unaffected (Fig. 7B). In antibody blocking assays, treatment with a GRP78-specific antibody dose-dependently reduced the titers of PEDV, TGEV, and SARS-CoV-2, while the other four viruses showed no significant changes (Fig. 7C). Together, these results establish GRP78 as a novel cofactor for coronavirus entry, albeit with variable dependency across different viruses. This finding deepens our understanding of coronavirus entry mechanisms and provides a theoretical basis for broad-spectrum antiviral strategies targeting GRP78.

## DISCUSSION

**Fig 8:**
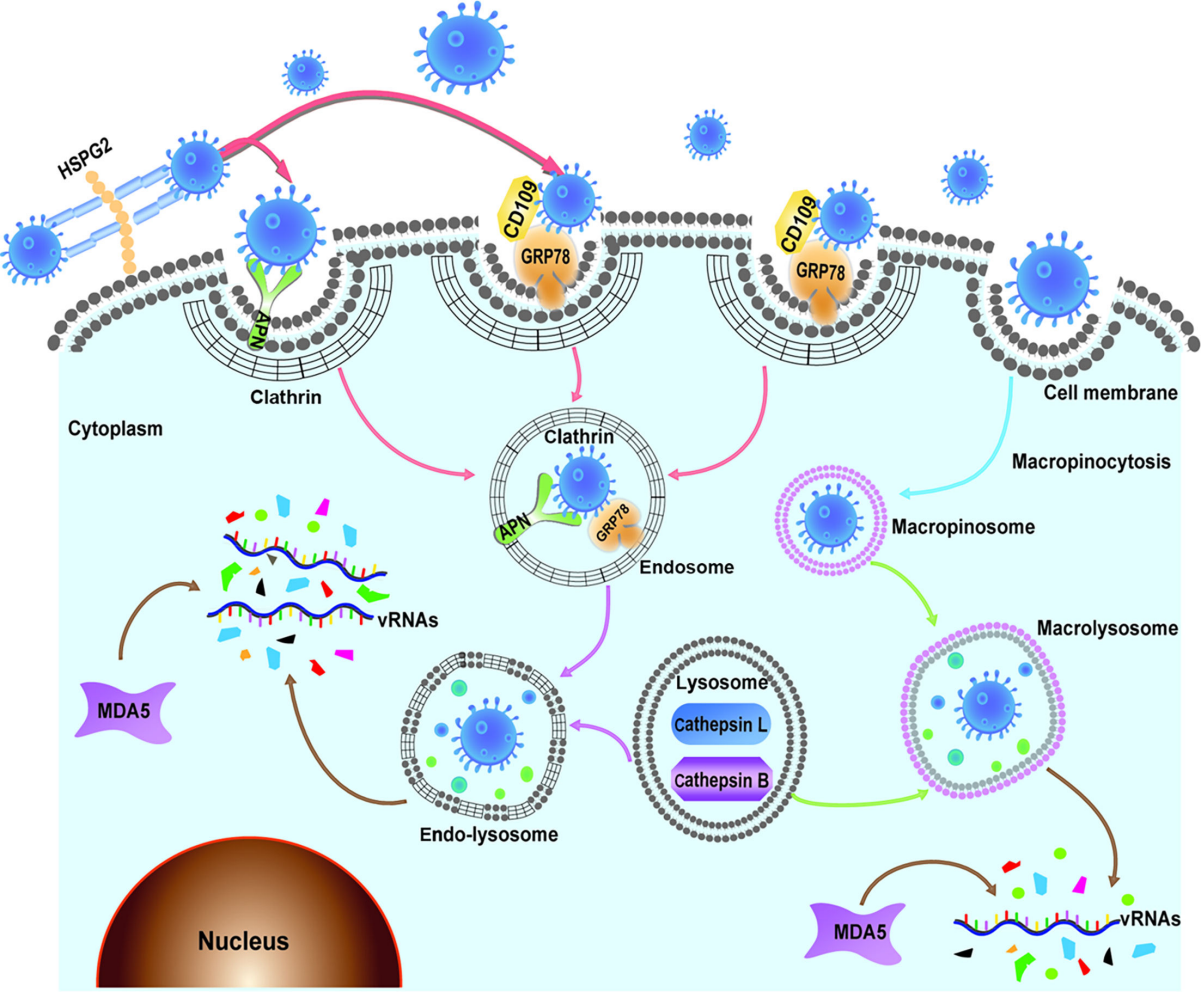
Hypothetical model of PDCoV entry into IPEC-J2 cells. First, PDCoV binds to the attachment receptor HSPG2. This initial docking step concentrates viral particles on the cell surface, allowing them to bind to the entry receptors pAPN and GRP78. Second, the virus is internalized via clathrin-mediated endocytosis (the primary pathway) or macropinocytosis (the secondary pathway). Third, PDCoV-containing endosomes/macropinosomes fuse with lysosomes. This allows lysosomal proteases, such as cathepsins L and B, to cleave the viral particles. Finally, the viral RNA is released into the cytoplasm for translation and replication.

The emergence of novel coronaviruses (CoVs), such as SARS-CoV, MERS-CoV, SARS-CoV-2, CCoV-HuPn-2018, and PDCoV, is a serious public health concern. The spillover of CoVs from natural animal hosts to humans has resulted in three global epidemics in the 21st century and continues to endanger human and animal health (52, 53). Coronaviruses typically utilize multiple host factors for viral infection. Among this, the host factors used by SARS-CoV-2 were the most extensively studied. Reports indicated that high-density lipoprotein (HDL), neuropilin-1 (NRP1), DC-SIGN, histamine receptor H1 (HRH1), AXL, TMEM106B, and others can facilitate SARS-CoV-2 entry (54–59). Similarly, previous studies suggested PDCoVs also could employ multiple host factors for viral entry. In addition to the documented APN, there are other unidentified host factors facilitating viral entry and replication (35–37). In this study, we identified the host factor GRP78 can interact with PDCoV S1 protein directly using a protein-protein interaction approach and promote PDCoV attachment and internalization via a pAPN-independent alternative mechanism. The schematic diagram of these findings is graphically summarized in Fig. 8.

Although GRP78 is predominantly localized to the endoplasmic reticulum (60), using multiple methods including cell surface biotinylation assays and high-resolution confocal imaging, we have demonstrated that a small fraction of GRP78 is indeed present on the surface of porcine intestinal epithelial cells. This fraction of cell surface GRP78 is sufficient to serve as an “anchor” for viral attachment or entry, interacting with the viral S protein and initiating subsequent internalization. This finding is consistent with reports from other investigators demonstrating the existence of “cell surface GRP78” in various cell types (49, 61). According to previous references described, this multifunctional protein interacts with various ligands, including α2-macroglobulin (A2M), voltage-dependent anion channel (VDAC), major histocompatibility complex class I (MHC-I), and DnaJ-like protein MTJ-1 (62–64), which validate our affinity purification-mass spectrometry results. The involvement of GRP78 in PDCoV attachment and entry provides important insights into the host range and zoonotic potential of the virus. For instance, the high conservation of GRP78 across species raises concerns regarding the ability of PDCoV to infect other mammals, including humans (62). Although current PDCoV strains are not highly virulent in humans, they may become more pathogenic through recombination with other coronaviruses or by acquiring virulence factors (65). Therefore, understanding the molecular mechanisms of PDCoV entry is paramount for the development of targeted antiviral strategies and vaccines. Referred to previous studies and our findings (Fig. 7), GRP78 may act as a pan-coronavirus host factor and also functions as a coreceptor for dengue virus (66) and is involved in the entry of the Japanese encephalitis virus (67). As a broad-spectrum anti-coronavirus target, however, its application scope must take into account inter-virus differences. The molecular basis for this differential dependency remains to be elucidated but may be related to variations in S protein structure, the use of alternative receptors, or differences in the entry pathways employed by different coronaviruses. Future studies comparing the structural features of S proteins from GRP78-sensitive vs -insensitive coronaviruses may provide valuable insights into the determinants of GRP78 dependency and inform the development of GRP78-targeted broad-spectrum antiviral strategies.

Through knockdown, antibody blockade, and decoy protein competition experiments in primary porcine intestinal epithelial cells, we have demonstrated that GRP78 also plays a critical role in PDCoV entry in a cell model that more closely approximates the physiological state. The consistent results obtained from GRP78 overexpression and knockdown experiments across multiple cell lines spanning different species and tissue origins, along with the positive correlation between cell surface GRP78 levels and viral infectivity, collectively establish GRP78 as a conserved host factor for PDCoV entry whose function is not restricted to specific cell lines or species contexts. These findings provide a more robust foundation for developing broad-spectrum antiviral strategies targeting GRP78.

Another notable host factor for PDCoV is pAPN, which is abundantly expressed in intestinal epithelial cells, including IPEC-J2 cells (35, 68, 69). The abundance of both GRP78 and pAPN on the surface of intestinal epithelial cells suggests that PDCoV may exploit these two host factors simultaneously and synergistically to promote viral entry. Although GRP78 has broad chaperone functions, our data show that its knockdown does not alter the expression or surface localization of the known receptor pAPN. Taken together with the biochemical evidence for direct interaction between GRP78 and the PDCoV S1 protein, as well as antibody blockade and soluble protein competition experiments, we conclude that the role of GRP78 in PDCoV entry is at least partially achieved through its function as a direct viral binding site, rather than primarily through regulating the expression or function of other receptors. These two proteins independently mediate viral entry, serving as two distinct targets for antiviral drugs. Coronavirus host factors typically interact with the well-characterized C-terminal domain (CTD) of the S1 subunit of the spike protein, which contains a receptor-binding domain (RBD) (70, 71). We demonstrated that the CTD of the PDCoV S1 subunit interacts with the substrate-binding domain (SBD) of GRP78. This provides mechanistic insights into how GRP78 facilitates PDCoV entry and points the way for research on small-molecule antiviral drugs. However, another porcine coronavirus, PEDV, which infects the same organ (the small intestine) in the same host organism, utilizes the NBD region of GRP78 for cell entry (49). This result underscores the diversity of entry mechanisms among coronaviruses and adds to the growing body of evidence indicating that coronaviruses have evolved distinct receptor-binding strategies to enter cells over the course of evolution. Furthermore, using specific clathrin and macropinocytosis inhibitors, our results suggest that PDCoV primarily enters host cells via clathrin-mediated endocytosis (Fig. 6), a pathway utilized by other CoVs, including infectious bronchitis virus (IBV) and PEDV (45, 49). This reliance on receptor-mediated endocytosis reveals potential therapeutic strategies for blocking PDCoV entry.

To confirm the robust role of GRP78 in PDCoV attachment and entry, we attempted to establish knockout cell lines using the CRISPR/Cas9 system. However, we could not generate IPEC-J2-GRP78^KO^ cells because GRP78 knockout resulted in high cell mortality. These observations align with previous studies that have demonstrated that GRP78 is essential for maintaining cellular homeostasis and survival (46, 47). Compared with previous studies, we expressed the GRP78 protein for *in vitro* interaction validation and affinity determination. This approach differs from earlier research, which was limited to verifying interactions through intracellular overexpression. Affinity experiments can intuitively assess the binding strength between two proteins, further clarifying the role of the GRP78 protein in viral invasion. Our findings show that the affinity between the GRP78 protein and the PDCoV S1 protein is greater than that between pAPN and PDCoV S1 (Fig. 4D and E). However, it is still in the micromolar range, which is characteristic of virus-attachment factor/coreceptor interactions. GRP78 may primarily function as an initial attachment factor, enriching the virus on the cell surface and thereby facilitating subsequent binding of the virus to pAPN. This mode of action is consistent with the functional characteristics of a classical “attachment factor” rather than those of an independent “primary receptor.” Therefore, we define GRP78 as a “critical host entry factor that promotes viral attachment and internalization” and propose that it may play a synergistic and sequential role with pAPN during viral entry.

In the present study, we identified a functional link between GRP78 and the clathrin-mediated endocytic pathway during PDCoV entry. Our study reveals a constitutive basal interaction between GRP78 and clathrin, which may reflect the physiological function of GRP78 as a chaperone involved in cellular endocytic trafficking. More importantly, PDCoV infection significantly enhances this interaction, and this enhancement depends on the binding of viral S1 to GRP78. This virus-specific recruitment event may represent a novel mechanism by which the virus, through binding to surface GRP78, induces its coupling to the endocytic machinery, thereby hijacking the clathrin-mediated endocytic pathway for efficient entry. This provides new insights into how coronaviruses coordinate host factors to facilitate entry.

Although GRP78 meets some criteria for a viral receptor (direct binding to the virus and essentiality for entry), its efficiency in mediating entry into non-permissive cells is lower than that of the primary receptor pAPN. Moreover, GRP78 is predominantly localized in the endoplasmic reticulum, with only a small fraction expressed on the cell surface. These characteristics suggest that GRP78 likely functions more as an “entry cofactor” or “coreceptor” facilitating viral attachment and internalization either in coordination with pAPN or independently, rather than serving as a conventional primary receptor. Future studies are needed to further elucidate the precise division of labor and cooperative mechanisms between GRP78 and pAPN during viral entry. Interestingly, in this study, we noted that different indicators have different sensitivities: viral titer detection is generally more sensitive than Western blot for detecting viral protein expression and can amplify differences in viral replication. Therefore, when evaluating the effects of an intervention, the combined use of multiple indicators can provide more comprehensive information.

Except for GRP78, our findings also show that HSPG2 and CD109 can act as co-factor in the entry of PDCoV. HSPG2 was reported as a pan-vial attachment host factor of several viruses, including human papillomavirus (HPV) (72), herpes simplex virus (HSV) (73), and dengue virus (74). CD109, another protein identified in our study, has also been suggested to function as a viral coreceptor. Recent studies have shown that CD109 can interact with viral proteins and facilitate viral entry in certain cell types (75). Furthermore, the involvement of CD109 in modulating TGF-β signaling may also contribute to viral pathogenesis (76). However, despite this evidence, the exact role of CD109 in PDCoV entry remains unknown and requires further investigation.

As with the majority of studies, there are three major limitations in this study that could be addressed or optimized in future research. First, as a key objective in viral entry research, we have made multiple attempts to resolve the structural basis of the interaction between the S1 protein and the cell membrane protein GRP78, but these efforts were ultimately unsuccessful. This difficulty is likely attributable to the intrinsic instability and conformational flexibility of the membrane protein, which pose significant challenges to structural determination. Second, the suboptimal quality of the extracted membrane proteins resulted in significant interference from cytoplasmic components, which affected the binding of the S1 protein to the membrane proteins. It is possible that other host factors promoting the PDCoV entry remain unidentified. In future studies, we should consult professor Zhang’s team on the method for extracting membrane proteins to better obtain key host factors (49). Third, due to the crucial role of GRP78 for cell survival, the construction of GRP78 knock-out cell line is unsuccessful. This prevents us from obtaining robust results and also makes animal experiments impossible. Similarly, other studies have yet failed to successfully construct GRP78 knockout cell lines (67, 77).

In conclusion, this study identifies GRP78 as a novel host factor that facilitates PDCoV attachment and internalization via clathrin-mediated endocytosis, operating independently of the canonical receptor pAPN. GRP78 directly interacts with the PDCoV S1 protein through its substrate-binding domain (SBD), and this interaction promotes viral entry. Notably, GRP78 also enhances the entry of several other coronaviruses, albeit with varying degrees of dependency. Together, these findings advance our understanding of the early entry mechanisms of PDCoV and highlight GRP78 as a conserved host factor involved in coronavirus attachment and internalization.

## MATERIALS AND METHODS

### Cells and viruses

Porcine intestinal epithelial cells (IPEC-J2), porcine kidney cells (LLC-PK1), porcine kidney-15 (PK15), human embryonic kidney 293T (HEK-293T), baby hamster kidney cells (BHK-21), VeroE6 cells, swine testis cells (ST), cellosaurus cell line 17Cl-1 cells, Huh7 cells, and Huh7-ACE2 cells were cultured in Dulbecco’s modified Eagle medium (DMEM; Gibco) supplemented with 10% fetal bovine serum (FBS; Gibco), 100 U/mL penicillin, and 0.1 mg/mL streptomycin (Sigma) at 37°C in a humidified incubator with 5% CO₂. Porcine primary small intestinal mucosal epithelial cells (PIG-iCell-d007) were cultured in specialized culture medium (#iCell-d007-002p) purchased from iCell Bioscience company (Shanghai, China). Sf9 insect cells were maintained in insect SF9/SF21 cell medium (Sino Biological, Inc., China) supplemented with 100 U/mL penicillin and 0.1 mg/mL streptomycin (Sigma) at 27°C. These cells were confirmed to be *Mycoplasma* free by using a PCR-based assay (57).

The PDCoV CHN-HN-1601, PEDV BJ-2011-C, SADS-CoV, TGEV, MHV-A59, HCoV-229E, and HCoV-NL63 strains used in this study were maintained in our laboratory (68, 78). The SARS-CoV-2 (D614G) and EG.5 viruses were stored and handled in the P3 facility of the Guangzhou Laboratory. VeroE6 cells were employed for the propagation of PEDV, SADS-CoV, SARS-CoV-2 (D614G), and SARS-CoV-2 EG.5. TGEV was cultured in ST cells, while MHV-A59 was propagated in 17Cl-1 cells. HCoV-229E and HCoV-NL63 were cultivated using Huh-ACE2 cells.

### Antibodies and chemicals

The PDCoV nucleocapsid protein mouse monoclonal antibody (1A3) was produced in our laboratory and used as previously described (68). The PDCoV spike protein S1 mouse monoclonal antibody (G10C2) was obtained in-house using hybridoma technology. The following antibodies were purchased from Abcam: the rabbit monoclonal antibody (#ab108615) against GRP78, the rabbit polyclonal antibody (#ab236283) against pAPN, and the rabbit monoclonal antibody (#ab255829) against heparan sulfate proteoglycans 2/perlecan (HSPG2). The mouse monoclonal antibody (#sc-271085) against CD109 was purchased from Santa Cruz Biotechnology. The clathrin heavy chain (P1663) antibody (#2410) was purchased from Cell Signaling Technology. Protease inhibitor cocktail (#P8340), HRP-conjugated goat anti-mouse IgG antibody (#AP181P), HRP-conjugated goat anti-rabbit IgG antibody (#AP187P), mouse monoclonal antibody (#H3663) against the HA tag, mouse monoclonal antibody (#05-724) against the Myc tag, rabbit monoclonal antibody (#SAB4301135) against the FLAG tag, HA15 inhibitor (#SML2118), clathrin pathway inhibitors Pitstop 2 (#SML1169) (79) and Dynasore (#D7693) (80), macropinocytosis inhibitors 5-(N, N-dimethyl)amiloride hydrochloride (EIPA) (#A4562), and cytochalasin (cyto D, #C2618) were all purchased from Sigma-Aldrich. Alexa Fluor 488-conjugated goat anti-mouse IgG (H + L) cross-adsorbed secondary antibody (#A-11001) and Alexa Fluor 568-conjugated goat anti-rabbit IgG (H + L) cross-adsorbed secondary antibody (#A-11011) were obtained from Thermo Fisher Scientific.

### Indirect immunofluorescence assay

The IFA was performed as previously described (68). Briefly, cells were fixed with 4% paraformaldehyde (Sigma) at room temperature for 15 min and then washed three times with phosphate-buffered saline (PBS). Permeabilization was achieved by incubating the cells in 0.03% Triton X-100 (Sigma) in PBS for 15 min, followed by three additional washes with PBS. The cells were then blocked with 5% bovine serum albumin (BSA) in PBS for 15 min and washed three times with PBS. Primary antibodies were applied, and the cells were incubated at 37°C for 1 h. Then, the cells were washed three times with PBST (PBS containing 0.05% Tween-20). Alexa Fluor 488- or 568-conjugated secondary antibodies were added, and the cells were incubated at 37°C for 1 h in the dark. After three washes with PBST, the cells were stained with DAPI (1 µg/mL) for 2 min at room temperature. Fluorescence was visualized using an Eclipse Ci-S fluorescence microscope (Nikon, Japan), and the fluorescence intensity of IFA was quantified using ImageJ software.

### Western blotting

Protein samples were mixed with 5× protein loading buffer containing 5% β-mercaptoethanol (#P1041, Solarbio, China) and denatured by boiling for 10 min. The denatured proteins were separated by sodium dodecyl sulfate‒polyacrylamide gel electrophoresis (SDS-PAGE) and transferred to 0.22 µm polyvinylidene fluoride (PVDF) membranes (Millipore, Sigma). The membranes were blocked with 5% skim milk in PBS for 2 h at room temperature, followed by three washes with PBS. Primary antibodies specific to the target proteins were applied to the membranes and incubated for 1 h at room temperature. After three washes with PBST (PBS containing 0.05% Tween-20), the membranes were incubated with HRP-conjugated goat anti-mouse or anti-rabbit secondary antibodies for 1 h at room temperature. Following three additional washes with PBST, the membranes were incubated with enhanced chemiluminescence (ECL) reagents (Pierce, Thermo Fisher) for 2 min in the dark. Protein bands were visualized using a chemiluminescence apparatus (ProteinSimple), and densitometry analysis was conducted using ImageJ software.

### Quantitative real-time PCR

Quantitative real-time PCR was applied for the detection of coronaviruses N gene copies. Briefly, the RNA genome of coronaviruses was extracted from supernatants and cells using the TaKaRa MiniBEST Viral RNA/DNA Extraction Kit (#9766, TaKaRa). cDNA was synthesized using the FastQuant RT Kit (with gDNase; #KR106, TIANGEN, China). Primers targeting the N gene were listed in Table 1. Standard positive plasmids containing the full-length N gene were serially diluted (10⁸ to 10¹ copies/µL) to generate a standard curve. Quantitative real-time PCR was performed using SYBR Select Master Mix (#4472919, Applied Biosystems, Thermo Fisher Scientific) with the following reaction mixture: 10 µL of 2× SYBR master mix, 0.4 µL of each primer (10 µM), 2 µL of the cDNA template or standard plasmid, and 7.2 µL of RNase-free water. The PCR conditions were as follows: 50°C for 2 min, 95°C for 2 min, and 40 cycles of 95°C for 15 s, 58°C for 15 s, and 72°C for 40 s. The number of copies in each sample was calculated using the formula *Y* = *AX* + *B*, where *Y* represents copies/µL, *A* is the slope of the standard curve, *X* is the Ct value, and *B* is the intercept.

**TABLE 1:** Primers designed for quantitative real-time PCR

Primer name	Sequence (5′−3′)
PDCoV N-F	AGGGTTCGGGAGCTGACACTTCT
PDCoV N-R	GGTCGCGTTTCCTGGGCTGATT
PEDV N-F	CACAGATAGTGAGAAAGTGCTTC
PEDV N-R	GTGGGTACAGCATTATTTGCAAG
SADS-CoV N-F	GGAATGTCATGCCTAGAAATGGAG
SADS-CoV N-R	GTGCCAGTTAGAAGGCTGATCTTTAC
MHV-A59 N-F	GCCAATGGAATCCCCGCTTC
MHV-A59 N-R	GAAGACACCTTCAATGCTGTC
HCoV-229E N-F	TGGCACAGGACCCCATAAAG
HCoV-229E N-R	CAACCCAGACGACACCTTCA
HCoV-NL63 N-F	GTCACCTAGTTCTTCTGGTACTTCCA
HCoV-NL63 N-R	GCTTATCAGCCCTGGGTTGA
SARS-CoV-2 N-F	AAGAAATTCAACTCCAGGCAGC
SARS-CoV-2 N-R	GCTGGTTCAATCTGTCAAGCAG
HSPG2 qPCR-1F	GTGGCCTCCTACGTCACCTC
HSPG2 qPCR-1R	CTCCAGGGCCACACACTCAT
CD109 qPCR-1F	TGACAGATGCAAACCTCACGAA
CD109 qPCR-1R	GCTTTCGGACATGTGGACTGC
pAPN qPCR-1F	AACTCCCTGCTGTTCGACCC
pAPN qPCR-1R	CCTCGTTCAGCCACAGGTCA
GRP78 qPCR-1F	CGCTGGAACTATTGCTGGC
GRP78 qPCR-1R	AGACACATCGAAGGTTCCGC
β-Actin qPCR-1F	TCGTGATGGACTCCGGTGAC
β-Actin qPCR-1R	GTAGTCAGTCAGGTCCCGGC

For relative real-time PCR for the detection of gene expression levels, cells were collected, and mRNA was extracted using TaKaRa MiniBEST Universal RNA extraction kit (#9767, TaKaRa). cDNA was synthesized using the FastQuant RT Kit. The primers targeting specific genes are listed in Table 1. Real-time PCR was performed using SYBR Select Master Mix with the following reaction mixture: 10 µL of 2× SYBR master mix, 0.4 µL of each forward and reverse primer (10 µM), 2 µL of the cDNA template, and 7.2 µL of RNase-free water. The PCR conditions were as follows: 50°C for 2 min, 95°C for 2 min, and 40 cycles of 95°C for 15 s, 58°C for 15 s, and 72°C for 40 s. The GAPDH gene was used as a control, and the relative gene expression levels were measured using the 2^-(△△ct)^ method.

### TCID_50_ assay

Due to the low replication efficiency of PDCoV in IPEC-J2 cells, we selected LLC-PK1 cells for viral titer determination. LLC-PK1 cells were seeded in 96-well plates and cultured in DMEM supplemented with 10% FBS until they reached 90%–100% confluence. Then, PDCoV-containing cell culture media and cell lysates were serially diluted 10-fold in DMEM containing 10 µg/mL trypsin and 37.5 µg/mL pancreatin. Next, 100 µL of each dilution was inoculated into the cells. After 72 h post-infection (hpi), the media were removed and the cells were fixed with 4% paraformaldehyde. Viral titers were determined using the Reed-Muench endpoint method, as described in the IFA assay section.

### Eukaryotic protein expression and purification

Recombinant proteins, including PDCoV-S1-linker(GGGGS)₃-Fc (human), PDCoV-S1-His, IPEC-J2-GRP78-linker(GGGGS)₃-Fc (human), and Fc (human), were expressed using the Bac-to-Bac baculovirus expression system. pBeloBAC11-PDCoV plasmids (68), GRP78 mRNA from IPEC-J2 cells, and the human Fc DNA sequence (GenBank accession no. BC092518, synthesized by TsingKE Biological Technology Company, Beijing, China) were used as templates to amplify PDCoV-S1, GRP78, and Fc DNA fragments, respectively. Primers (listed in Table 2) were synthesized by TsingKE Biological Technology Company. DNA fragments for S1-Fc and GRP78-Fc were constructed via homologous recombination using the NEBuilder DNA Assembly Kit (#E2621S, NEB). The fragments were ligated into the pFastBac Dual donor vector, which was double-digested with *BamH*I and *EcoR*I (NEB), and transfected into DH5α *E. coli* cells. Positive recombinant plasmids were confirmed by PCR and sequencing and then transfected into DH10Bac *E. coli* cells containing a Bacmid and helper plasmid. Positive colonies were selected via blue/white screening on LB agar plates containing 50 µg/mL kanamycin (#E004000, Sigma), 7 µg/mL gentamicin (#E003632, Sigma), 10 µg/mL tetracycline (#58346-M, Sigma), 100 µg/mL X-gal (#71077, Sigma), and 40 µg/mL IPTG (#I6758, Sigma). The recombinant bacmids were isolated using the S.N.A.P. MidiPrep Kit (#K1910-01, Invitrogen) and verified by PCR using M13 forward (−40) and reverse primers (forward: 5′-GTTTTCCCAGTCACGAC-3′; reverse: 5′-CAGGAAACAGCTAGAC-3′) and Platinum Taq DNA polymerase (#15966005, Invitrogen). The expected PCR product size was ~2,560 bp plus the size of the inserted DNA fragment. The recombinant bacmids were transfected into Sf9 cells cultured in insect SF9/SF21 medium without antibiotics using the Cellfectin II reagent (#10362100, Invitrogen). After 72 h, the cell culture medium was harvested and centrifuged at 6,000 × *g* for 5 min to obtain the P1 viral stock. The viral titer was amplified by serial passaging. Protein expression was confirmed by IFA and WB. For large-scale protein production, P4 viral stocks were used to infect Sf9 cells in 500 mL flasks (160 rpm, 27°C) at an MOI of 1 for 96 h. Cells were harvested by centrifugation at 6,000 × *g* for 10 min and lysed in NP-40 buffer (#P0013F, Beyotime, China) supplemented with protease inhibitor cocktail. Supernatants were collected for protein purification.

**TABLE 2:** Primers designed for constructing recombinant plasmids

Primer name	Sequence (5′−3′)
Linker-Fc-F	GGAGGAGGAGGATCAGGAGGAG
Linker-Fc-R	TAGTACTTCTCGACAAGCTT TTATCATTTACCCGGAG
pFastBac Dual-Fc-F	CATCGGGCGCGGATC ATGGGAGGAGGAGGATCAGGA
pFastBac Dual-Fc-R	TAGTACTTCTCGACAAGCTT TTATCATTTACCCGGAG
pFastBac Dual-S1-Fc-F	CCCACCATCGGGCGCGGATC ATGCAGAGAGCTCTATTGAT
pFastBac Dual-S1-Fc-R	TCCTGATCCTCCTCCTCC TATTTCAACTTCGCCATCGT
pFastBac Dual-S1-his-F	CCCACCATCGGGCGCGGATC ATGCAGAGAGCTCTATTGAT
pFastBac Dual-S1-his-R	GGCGAATTCTTAGTGGTGGTGGTGGTGGTGTATTTCAACTTCGCCATCGT
pFastBac Dual-GRP78-Fc-F	CCCACCATCGGGCGCGGATC ATGAAGCTGTCCCTG
pFastBac Dual-GRP78-Fc-R	TCCTGATCCTCCTCCTCC CTACAACTCATCTTTG
pCAGGS-HA-GRP78-F	GTTCCAGATTACGCTGAATTCATGAAGCTGTCCCTG
pCAGGS-HA-GRP78-R	ATTAAGATCTGCTAGCTACAACTCATCTTTG
pCAGGS-HA-pAPN-F	GTTCCAGATTACGCTGAATTCATGGCCAAGGGCTTC
pCAGGS-HA-pAPN-R	ATTAAGATCTGCTAGCTCGAGCTATTTGCTGTTTTCTG
pCAGGS-HA-CD109-F	GTTCCAGATTACGCTGAATTCATGCATATGCGTTTG
pCAGGS-HA-CD109-R	ATTAAGATCTGCTAGTTAGAAAGCAAATCC
p3 × Flag-CMV-10-S1-F	ccgGAATTCAATGCAGAGAGCTCTATTG
p3 × Flag-CMV-10-S1-R	cgcGGATCCTTATATTTCAACTTCGCCATCGT
pBiFC-VN173-S1-F	CCGGAATTCGGATGCAGAGAGCTCTATTG
pBiFC-VN173-S1-R	CCGCTCGAGATATTTCAACTTCGCCATCGT
pBiFC-VC155-GRP78-F	ATGGAGGCCCGAATTCGG ATGAAGCTGTCCCTG
pBiFC-VC155-GRP78-R	CGGACGGGTACCTCGAGA CTACAACTCATCTTTG
pBiFC-VC155-pAPN-F	ATGGAGGCCCGAATTCGG ATGGCCAAGGGATTC
pBiFC-VC155-pAPN-R	CGGACGGGTACCTCGAGA CTATTTGCTGTTTTCTG
pCMV-Myc-NTD-F	GCCATGGAGGCCCGAATTCGGATGtttgatgttggcgttc
pCMV-Myc-NTD-R	GCGGCCGCGGTACCTCGAGCTACTCCACTTCGTGagaagag
pCMV-Myc-CTD-F	GCCATGGAGGCCCGAATTCGGATGCCTGAGCTTGAAGTA
pCMV-Myc-CTD-R	GCGGCCGCGGTACCTCGAGCTATGTTGCATGTACTGA
pCAGGS-HA-NBD-F	CCAGATTACGCTGAATTC ATGCCATATATTCAAGTTG
pCAGGS-HA-NBD-R	TTAATTAAGATCTGCTAG CTATTTCCGAACATCTTTG
pCAGGS-HA-SBD-F	CCAGATTACGCTGAATTC ATGCCCCTTACACTTGG
pCAGGS-HA-SBD-R	TTAATTAAGATCTGCTAG CTAGGTGACTTCAATC
pCAGGS-HA-V-F	CCAGATTACGCTGAATTC ATGTTTGAAATAGATGTGA
pCAGGS-HA-V-R	TTAATTAAGATCTGCTAG CTAGTCTGCTGATTCCTC

The recombinant proteins (S1-Fc, GRP78-Fc, N-Fc, Fc, S1-His, GRP78-His, and N-His) were purified using Pierce Protein A/G magnetic beads (#88802, Thermo Fisher Scientific) and Ni-NTA agarose (#30210, QIAGEN) respectively, following the manufacturers’ protocols. After affinity purification, the proteins were further isolated using ENrich SEC650 high-resolution size exclusion columns (#780-1650, Bio-Rad) on an NGC chromatography system. Protein purity was assessed by SDS-PAGE and WB.

### Plasma membrane profiling

Plasma membrane profiling was performed as previously described (81), using the Pierce Cell Surface Protein Biotinylation and Isolation Kit (#A44390, Thermo Fisher Scientific). Minor modifications to the protocol were introduced for adherent cells. Briefly, IPEC-J2 cells grown in a 15 cm dish (>85% confluency) were washed twice with 20 mL of sterile PBS. The PBS was promptly aspirated, and the cells were labeled with 10 mL of Sulfo-NHS-SS-Biotin (dissolved in PBS) for 10 min at room temperature. After removing the labeling solution, the cells were washed twice with ice-cold TBS, scraped into 10 mL of ice-cold TBS, and centrifuged at 500 × *g* for 3 min at 4°C. The supernatant was discarded, and the cell pellet was lysed in a buffer containing protease inhibitors and 1% Triton X-100. The lysate was incubated on ice for 30 min, followed by centrifugation at 15,000 × *g* for 3 min at 4°C. Next, 250 µL of NeutrAvidin Agarose slurry was added to the clarified supernatant and incubated for 30 min at room temperature with end-over-end mixing on a rotator. After three washes with washing buffer, the agarose beads were resuspended in 200 µL of elution buffer containing 10 mM DTT and incubated for 30 min at room temperature. The eluate containing plasma membrane proteins was collected by centrifugation at 1,000 × *g* for 2 min. Protein purity was confirmed by Western blot analysis of β-actin levels.

### Co-IP and LC-MS/MS

For identification of PDCoV-S1-interacting proteins, the purified S1-Fc protein was incubated with Protein A/G magnetic beads at 37°C for 1 h, after which the beads were collected using a magnetic separator. The beads were then washed three times with a washing buffer (10 mM phosphate buffer, 150 mM NaCl, pH 7.4). Plasma membrane proteins were pre-cleared with Protein A/G beads to remove nonspecific binding. Then, they were mixed with S1-Fc-bound beads and incubated at 4°C overnight. The beads were washed three times, and the bound proteins were eluted with IgG elution buffer (0.1 M glycine, pH 2.0–3.0). The eluted proteins were send to Proteomics and Metabolomics Facility of Guangzhou National Laboratory and identified by mass spectrometry (MS).

For interaction between GRP78-Fc and PDCoV-S1-His (or PDCoV-N-His), purified GRP78-Fc proteins were incubated with Protein A/G magnetic beads at 37°C for 1 h. After washing, PDCoV-S1-His or PDCoV-N-His (negative control) proteins that had been pre-cleared with Protein A/G beads were added and incubated at 4°C overnight. Afterward, beads were washed, and eluted proteins were analyzed by WB with specific tag antibodies.

For interaction between GRP78-His and PDCoV-S1-Fc (or PDCoV-N-Fc), purified PDCoV-S1-Fc proteins or PDCoV-N-Fc proteins (negative control) were incubated with Protein A/G magnetic beads at 37°C for 1 h. After washing, GRP78-His proteins that had been pre-cleared with Protein A/G beads were added and incubated at 4°C overnight. Afterward, beads were washed, and eluted proteins were analyzed by WB.

For binding domain analysis, the GRP78 protein was divided into three domains: NBD (125–280 aa), SBD (420–500 aa), and variable domain (115–650 aa). The PDCoV-S1 protein was divided into two domains: NTD (52–277 aa) and CTD (302–422 aa). Primers for constructing pCMV-Myc-NTD, pCMV-Myc-CTD, pCAGGS-HA-NBD, pCAGGS-HA-SBD, and pCAGGS-HA-V are listed in Table 2. DNA fragments were amplified using Platinum SuperFi II DNA polymerase and ligated into pCMV-Myc or pCAGGS-HA vectors using NEBuilder HiFi DNA Assembly Master Mix. Positive plasmids were extracted using the PureYield Plasmid Maxiprep System (#A2392, Promega) and transfected into BHK-21 cells. At 36 hpi, cells were lysed in NP-40 buffer containing protease inhibitor cocktail. Lysates were centrifuged at 12,000 rpm for 15 min at 4°C, and supernatants were collected. HA-tag antibody and mouse IgG (isotype control) were incubated with Protein A/G magnetic beads at 37°C for 1 h. Beads were washed three times and used for Co-IP.

For interaction between GRP78, clathrin, and pAPN, IPEC-J2 cells were transfected with a pCAGGS-HA-GRP78 recombinant plasmid. After 48 h, the cells were lysed in NP-40 buffer containing a protease inhibitor cocktail. The lysates were centrifuged at 12,000 × *g* for 15 min at 4°C, and the supernatants were collected. To minimize nonspecific binding, the supernatants were pre-cleared with Protein A/G beads. The pre-cleared lysates were then incubated overnight at 4°C with gentle rotation using anti-HA-conjugated beads. Following incubation, the beads were washed three times with lysis buffer, and the bound proteins were eluted with IgG elution buffer. The eluted proteins were subjected to Western blot analysis, where GRP78, clathrin, and pAPN were detected using anti-HA, anti-clathrin, and anti-pAPN antibodies, respectively.

### Specific antibody blocking assays

IPEC-J2 cells were cultured in 12-well plates and treated with 100 µg/mL of antibody anti-HSPG2, anti-GRP78, anti-pAPN, anti-CD109, or mouse IgG (isotype control) to block the corresponding proteins on the cell surface. The cells were then incubated at 37°C for 2 h in a humidified incubator with 5% CO₂. DMEM was used as a negative control. After blocking, the cells were washed three times with sterile PBS and inoculated with PDCoV at an MOI of 0.1 for 1.5 h at 37°C. Following three additional washes, 2 mL of DMEM containing 5 µg/mL of trypsin was added to the cells. At 24 hpi, the cells were fixed with 4% paraformaldehyde for IFA observation. ImageJ software was used to measure the fluorescence intensity. Additionally, cell culture supernatants and cells were collected, and viral titers and genome copies were quantified using the TCID₅₀ assay and real-time RT-PCR, respectively.

### RNAscope *in situ* hybridization

To quantify the number of PDCoV RNA copies attached to IPEC-J2 cells, RNAscope *in situ* hybridization was performed. Briefly, IPEC-J2 cells in 12-well plates were washed three times with sterile PBS and blocked with either anti-GRP78, anti-pAPN, or both antibodies, along with mouse IgG (isotype control), at 37°C for 2 h. DMEM served as a negative control. After blocking, the cells were inoculated with PDCoV at an MOI of 1 for 2 h at 4°C. Then, the cells were washed and fixed with 10% formaldehyde. The RNAscope Multiplex Fluorescent Reagent Kit v2 (Advanced Cell Diagnostics Inc., Hayward, CA, USA) was used according to the manufacturer’s instructions with 20 double-Z branched-pair probes (lot 17208B) targeting the PDCoV N gene. Fluorescence signals were visualized using a fluorescence microscope (ECLIPSE Ci-S, Nikon, Japan), and fluorescence intensity was calculated using ImageJ software. The PDCoV N gene copy number was measured by quantitative real-time RT-PCR.

### Plasmid overexpression

Recombinant eukaryotic expression plasmids were constructed using the primers listed in Table 2. pCAGGS-HA-GRP78-Mutant plasmids which were resistant to siGRP78 were constructed by Tsingke Biotechnology Co., Ltd (Beijing, China) and verified by DNA sequencing. BHK-21 cells in 12-well plates were transfected with 1 µg of plasmid DNA using Lipofectamine LTX with PLUS Reagent (#15338030, Thermo Fisher Scientific). At 48 h post-transfection, the cells were washed three times with sterile PBS and inoculated with PDCoV at an MOI of 1 for 1.5 h at 37°C. After washing, 2 mL of DMEM containing 5 µg/mL trypsin was added. At 24 hpi, the cell culture supernatant and cells were collected for IFA detection, real-time RT‒PCR, and TCID₅₀ assays. ImageJ software was used to measure the fluorescence intensity of IFA images.

### RNA interference

Small interfering RNAs (siRNAs) were designed and synthesized by GenePharma Biological Company (Suzhou, China). Their sequences are listed in Table 3. To silence a single gene, 20 pmol of siRNA was mixed with 6 µL of Lipofectamine RNAiMAX (#13778150, Thermo Fisher Scientific) and then transfected into IPEC-J2 cells in 12-well plates. GAPDH-targeting siRNAs were used as a positive control for gene silencing, and non-targeting siRNAs were used as a negative control. At 6 h post-transfection, the media were replaced with fresh DMEM. Forty-eight hours post-transfection, the cells were inoculated with PDCoV at an MOI of 0.1. At 24 hpi, the cell culture medium and cells were collected to determine the viral titer. For double gene silencing, 10 pmol of siRNA for each gene was used, and the same procedure was followed. Target gene expression levels were measured by relative real-time RT-PCR and WB assay.

**TABLE 3:** siRNAs designed for target gene knockdown

siRNA name	Sequence (5′−3′)
siRNA-HSPG2-1	UAUUAAGAAAGAUCAAGACAAdTdT
siRNA-HSPG2-2	UGUGUUUGUGGAUACACAGCGdTdT
siRNA-CD109-1	AUUGAUAAACCAUGAUUUCCUdTdT
siRNA-CD109-2	UCUUGAAAGAGCCUUUUUCAAdTdT
siRNA-pAPN-1	ACAUUCAGGGCAUACAUGCCAdTdT
siRNA-pAPN-2	ACAUUGAGGCAAUCCAUUGGAdTdT
siRNA-GRP78-1	UGAAGAACUCUUUAACCAGUUdTdT
siRNA-GRP78-2	AUGGUUUAGUUUUCUUCUCAAdTdT
siRNA-NC	UUCUCCGAACGUGUCACGUTT
siRNA-GAPDH-PC	ACAUGUAGACCAUGUAGUGGA

### CRISPR/Cas9 technology

Single-guide RNAs (sgRNAs) targeting HSPG2, pAPN, and GRP78 were designed using E-CRISP and cloned into the PX459 vector (#62988, Addgene) via homologous recombination following double digestion with *Age*I and *EcoR*I. The sgRNA sequences are listed in Table 4. IPEC-J2 cells in 12-well plates were transfected with 1 µg of each sgRNA plasmid. After 24 h, the cells were selected with 1 µg/mL puromycin (#540411, Sigma) for an additional 48 h. The surviving cells were harvested, and indel mutations were detected using the Surveyor Nuclease Assay. Clonal cell lines were established by limited dilution, with approximately 60 cells plated in 10 mL of DMEM per 96-well plate. Knockout clones were confirmed by sequencing and WB analysis.

**TABLE 4:** sgRNAs for establishing target gene knockout cell lines

sgRNA name	Sequence (5′−3′)
sgRNA-HSPG2-1F	CACCGGGATCCTGGTGTTCAGCGG
sgRNA-HSPG2-1R	AAACCCGCTGAACACCAGGATCCC
sgRNA-HSPG2-2F	CACCCCTAGACAGGAAGGAAGTTC
sgRNA-HSPG2-2R	AAACGAACTTCCTTCCTGTCTAGG
sgRNA-pAPN-1F	CACCGGGCTCCTGGCAGATGAAG
sgRNA-pAPN-1R	AAACCTTCATCTGCCAGGAGCCC
sgRNA-pAPN-2F	CACCGATGTTGAACGTGGCCTTCA
sgRNA-pAPN-2R	AAACTGAAGGCCACGTTCAACATC
sgRNA-GRP78-1F	CACCGGCACCACCTACTCGTGCGT
sgRNA-GRP78-1R	AAACACGCACGAGTAGGTGGTGCC
sgRNA-GRP78-2F	CACCGTTCAGGCTGGTGTACTCTC
sgRNA-GRP78-2R	AAACGAGAGTACACCAGCCTGAAC

### Protein co-localization analysis

For co-localization of endogenous pAPN/GRP78 with spike protein in PDCoV-infected IPEC-J2 cells, the IPEC-J2 cells were inoculated into a 12-well cell culture plate at a density of 5 × 10^5^ cells/well. Once the cells reached 90% confluency, they were inoculated with PDCoV at an MOI of 0.1 and incubated for 24 h. Then, the culture medium was removed, and the cells were washed three times with PBS. After that, the cells were subjected to IFA with or without cell permeabilization. Images were captured using a Nikon A1 confocal microscope.

For co-localization of overexpressed proteins, the following combinations of recombinant plasmids (1 µg each) were used for transient transfection: pCAGGS-HA-GRP78 + p3×Flag-CMV-10-S1, pCAGGS-HA-pAPN + p3×Flag-CMV-10-S1, pCMV-Myc-S1-NTD + pCAGGS-HA-GRP78-NBD, pCMV-Myc-S1-NTD + pCAGGS-HA-GRP78-SBD, pCMV-Myc-S1-NTD + pCAGGS-HA-GRP78-V, pCMV-Myc-S1-CTD + pCAGGS-HA-GRP78-NBD, pCMV-Myc-S1-CTD + pCAGGS-HA-GRP78-SBD, and pCMV-Myc-S1-CTD + pCAGGS-HA-GRP78-V. These plasmids were transfected into BHK-21 cells using Lipofectamine LTX Reagent with PLUS Reagent (#15338030, Thermo Fisher Scientific), following the manufacturer’s instructions. Twenty-four hours post-transfection, the media were removed, and the cells were washed with PBS and fixed with 4% paraformaldehyde. Anti-HA tag, anti-Flag tag, and anti-Myc tag antibodies were used at a dilution of 1:5,000 for IFA as described above. The immunofluorescence-stained cells were examined using a Nikon A1 confocal microscope.

### Bimolecular fluorescence complementation assays

To confirm the interaction between GRP78 and PDCoV-S1, the following plasmids were obtained from Addgene: pBiFC-VN173 (#22010), pBiFC-VC155 (#22011), pBiFC-bjunVN173 (#22012), pBiFC-bFosVC155 (#22013), and pBiFC-bFos(deltaZIP)VC155 (#22014). pBiFC-VC155-GRP78∆SBD and pBiFC-VN173-S1∆CTD plasmids were constructed by Tsingke Biotechnology Co., Ltd (Beijing, China) and verified by DNA sequencing. The primers designed for constructing pBiFC-VN173-S1, pBiFC-VC155-GRP78, and pBiFC-VC155-pAPN are listed in Table 2. The GRP78, pAPN, and PDCoV-S1 DNA fragments were amplified using Platinum SuperFi II DNA polymerase and ligated into vectors via homologous recombination, following the NEBuilder HiFi DNA Assembly Master Mix manual instructions. Positive plasmids were confirmed by sequencing and extracted using the PureYield Plasmid Maxiprep System. Each plasmid was then transfected into BHK-21 cells, and the target protein expression was confirmed by IFA using anti-Myc tag or anti-HA tag antibodies. Next, pBiFC-VN173-S1 + pBiFC-VC155-GRP78, pBiFC-VN173-S1 + pBiFC-VC155-pAPN, pBiFC-VN173-S1 + pBiFC-VC155-GRP78∆SBD, pBiFC-VC155-GRP78 + pBiFC-VN173-S1∆CTD, and pBiFC-VC155-GRP78∆SBD + pBiFC-VN173-S1∆CTD were cotransfected into BHK-21 cells cultured in a 12-well plate, respectively. Cotransfection of cells with pBiFC-bjunVN173 and pBiFC-bFosVC155 (1 µg of each) served as a positive control. Cotransfection with pBiFC-bjunVN173 and pBiFC-bFos(deltaZIP)VC155 (1 µg of each) served as a negative control. Green fluorescence indicated interaction between the two proteins.

### Surface plasmon resonance

The binding affinities of GRP78 and PDCoV S1 were determined by surface plasmon resonance with a Biacore 8K^+^ instrument referred to previous studies described (11, 57). The binding affinity of pAPN for S1 was also monitored as a positive control. Briefly, GRP78 or pAPN proteins were immobilized on the CM5 sensor chips (GE Healthcare) via an amine coupling kit. PDCoV S1 proteins were serially diluted in HEPES buffer and followed over the chips. Response units (RUs) were calculated by subtracting responses of the reference channel (FC1) from responses of the active channel (FC2). Adjusted RUs were fitted to a 1:1 binding model utilizing Biacore insight evaluation software version 4.0.8.19879 (Cytiva). Both the association rate (“on rate,” Ka) and dissociation rate (“off rate,” Kd) were measured and analyzed. The KD value was calculated by dividing Ka by Kd (Kd/Ka). All experiments were performed in at least three independent replicates to ensure reproducibility.

### Bio-layer interferometry

The binding affinity between PDCoV-S1 and GRP78 or pAPN was determined using the BLI technique on an Octet R8 system (Sartorius, Germany). All assays were performed at 25°C in a standard assay buffer (PBS, pH 7.4, supplemented with 0.02% Tween-20 and 0.1% bovine serum albumin) to minimize nonspecific interactions. Briefly, protein A biosensors were hydrated in the assay buffer for at least 10 min prior to use. The sensors were then loaded with PDCoV-S1-Fc at a fixed concentration (20 μg/mL) until a stable baseline signal was achieved. After establishing a baseline in the assay buffer, sensors were exposed to a series of increasing concentrations of GRP78-His ranging from 2 μM to 128 μM and pAPN-His ranging from 8 μM to 512 μM in the assay buffer. Each concentration was tested in separate wells. Association kinetics were monitored for 90 s, followed by a dissociation step in the assay buffer for 120–300 s to assess the dissociation rate. A reference well containing assay buffer only was included to correct for any signal drift or nonspecific binding. The resulting binding sensorgrams were processed by subtracting the reference signal and aligning the baseline. Global fitting of the association and dissociation phases was performed using a 1:1 binding model provided by the instrument’s data analysis software, yielding the equilibrium dissociation constant (KD), association rate constant (ka), and dissociation rate constant (kd). All experiments were performed in at least three independent replicates to ensure reproducibility.

### Protein-binding domain prediction

Structural information for GRP78 (PDB ID 6EOB) and PDCoV S (PDB ID 6B7N) was obtained from the Protein Data Bank (PDB). Binding domains between PDCoV-S1 and GRP78 were predicted using PyMOL software (https://pymol.org/2/).

### Cell viability assay

Cell viability was measured using a Cell Counting Kit-8 (#C0038, Beyotime, Shanghai, China) according to the kit instructions. Cells were seeded in 96-well plates and treated with the indicated siRNAs for 48 h or inhibitors for 24 h at a gradient concentration at 37°C. Then, the cells were added with 10 µL of CCK-8 solution per well and cultured for another 1 h. The optical density at 450 nm (OD_450_) was recorded with a microplate reader (Varioscan LUX, Thermo Scientific Fisher).

### Inhibition of clathrin-mediated endocytosis and macropinocytosis

IPEC-J2 cells in 12-well plates were treated with Pitstop 2 (10 µM or 20 µM) or Dynasore (80 µM or 160 µM) for 1 h at 37°C to inhibit clathrin-mediated endocytosis. Then, the cells were inoculated with PDCoV at an MOI of 0.1 for 24 h. Similarly, IPEC-J2 cells were treated with EIPA (40 µM or 60 µM) or cytochalasin D (Cyto D; 4 µM or 8 µM) to inhibit macropinocytosis prior to PDCoV inoculation. Viral titers in the cell culture supernatant and cells were determined. Drug cytotoxicity was measured using the Cell Counting Kit-8 according to the manufacturer’s instructions.

### Authentic virus infection assay

All authentic virus-related experiments, including infection, RNA extraction, and quantification, were performed at the biosafety level 3 (BSL-3) facility of Guangzhou National Laboratory.

### Statistical analysis

Data from three independent experiments were analyzed using GraphPad Prism version 8.0 (La Jolla, CA, USA). An unpaired Student’s *t*-test was used to assess differences between groups. Statistical significance is indicated as follows: ns (not significant), *P < 0.05, **P < 0.01, and ***P < 0.001. Error bars represent the mean ± standard deviation (SD). ImageJ 1.8.0 (National Institutes of Health, USA) software was used to quantify WB band and fluorescence intensity.

## Data Availability

The data that support the findings of this study are available on request from the corresponding author.

## SUPPLEMENTAL MATERIAL

Fig. S1 jvi.00717-26-s0001.tif Detection of target gene expression knockdown or knockout.

Fig. S2 jvi.00717-26-s0002.tif Detection of cytotoxicity, antibody validation, GRP78 localization, and recombinant plasmid expression.

Fig. S3 jvi.00717-26-s0003.tif Effects of clathrin/macropinocytosis inhibitors on PEDV infection and GRP78 knockdown on pAPN/GRP78 expression.

Supplemental legends jvi.00717-26-s0004.docx Descriptive legends for supplemental material.

Table S1 jvi.00717-26-s0005.xlsx Results of LC-MS/MS analysis of host proteins co-immunoprecipitated with PDCoV S1.

ASM does not own the copyrights to Supplemental Material that may be linked to, or accessed through, an article. The authors have granted ASM a non-exclusive, world-wide license to publish the Supplemental Material files. Please contact the corresponding author directly for reuse.
