# Supplementary figures and images for: Identification of GRP78 as a novel host factor that facilitates zoonotic porcine deltacoronavirus internalization and replication via clathrin-mediated endocytosis
Source: J Virol. 2026 Jul 2;100(7):e00717-26. doi: 10.1128/jvi.00717-26 (PMC13386991; doi:10.1128/jvi.00717-26)

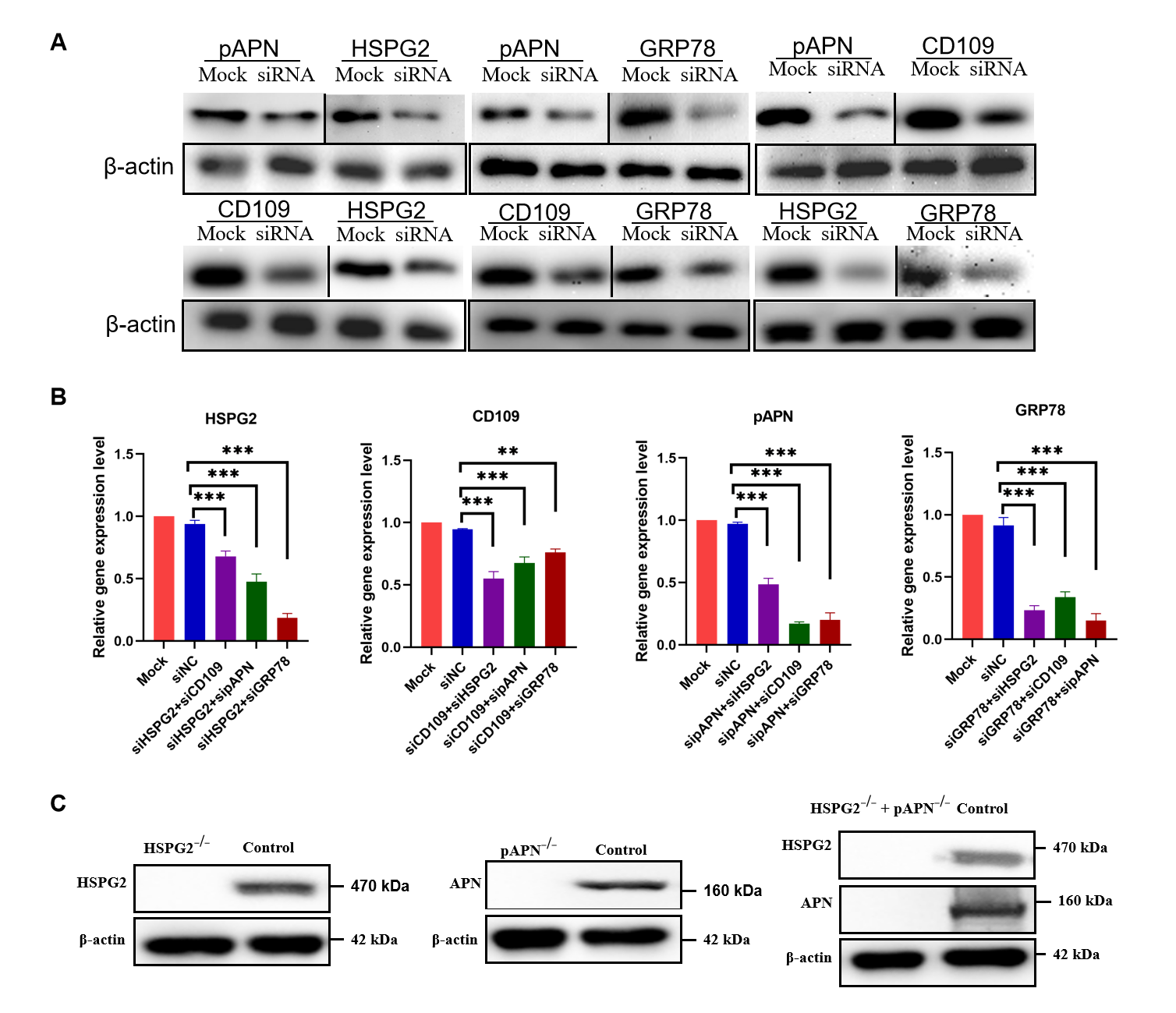

Supplement: Fig. S1 — Detection of target gene expression knockdown or knockout. [file jvi.00717-26-s0001.tif]

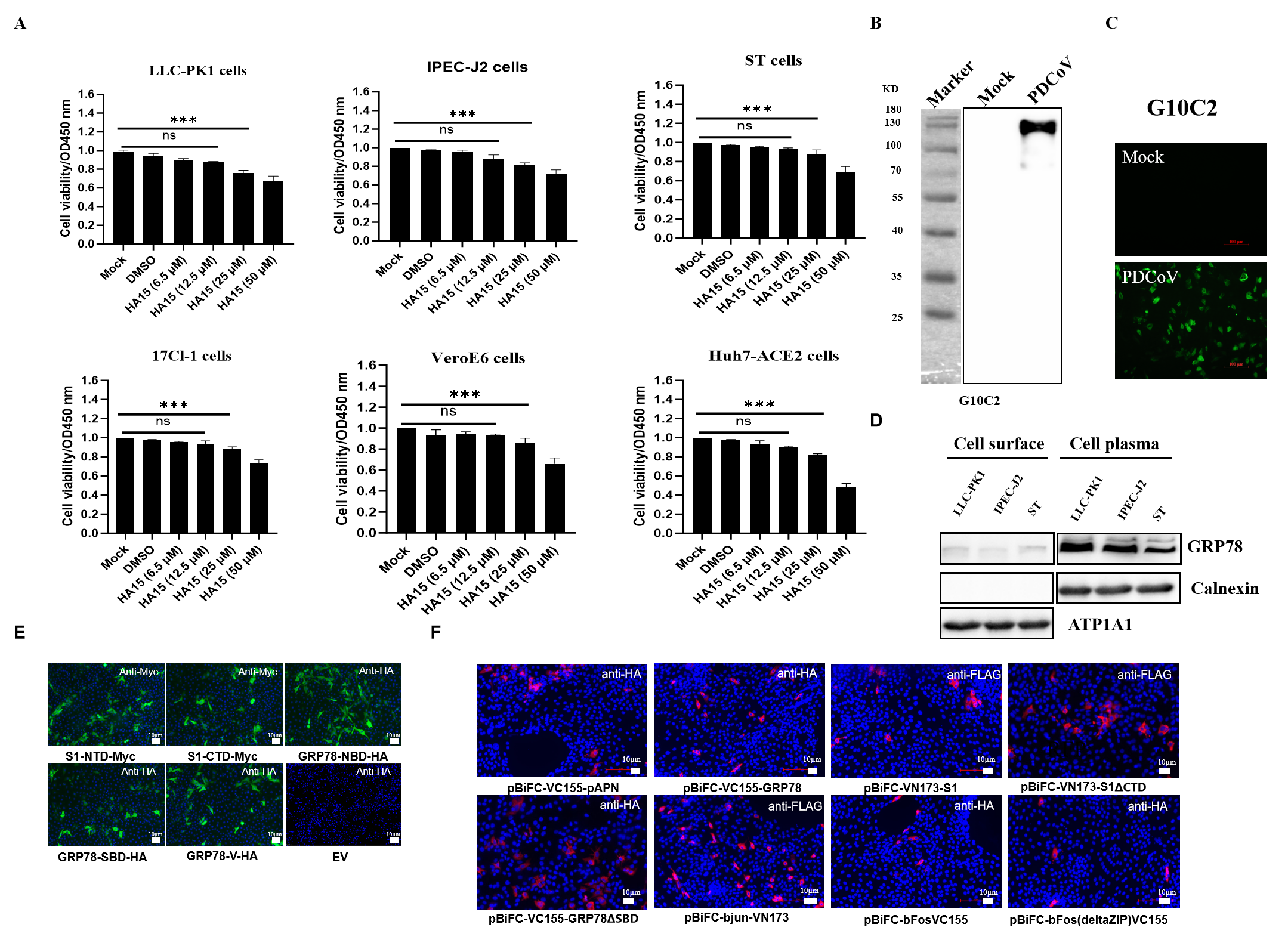

Supplement: Fig. S2 — Detection of cytotoxicity, antibody validation, GRP78 localization, and recombinant plasmid expression. [file jvi.00717-26-s0002.tif]

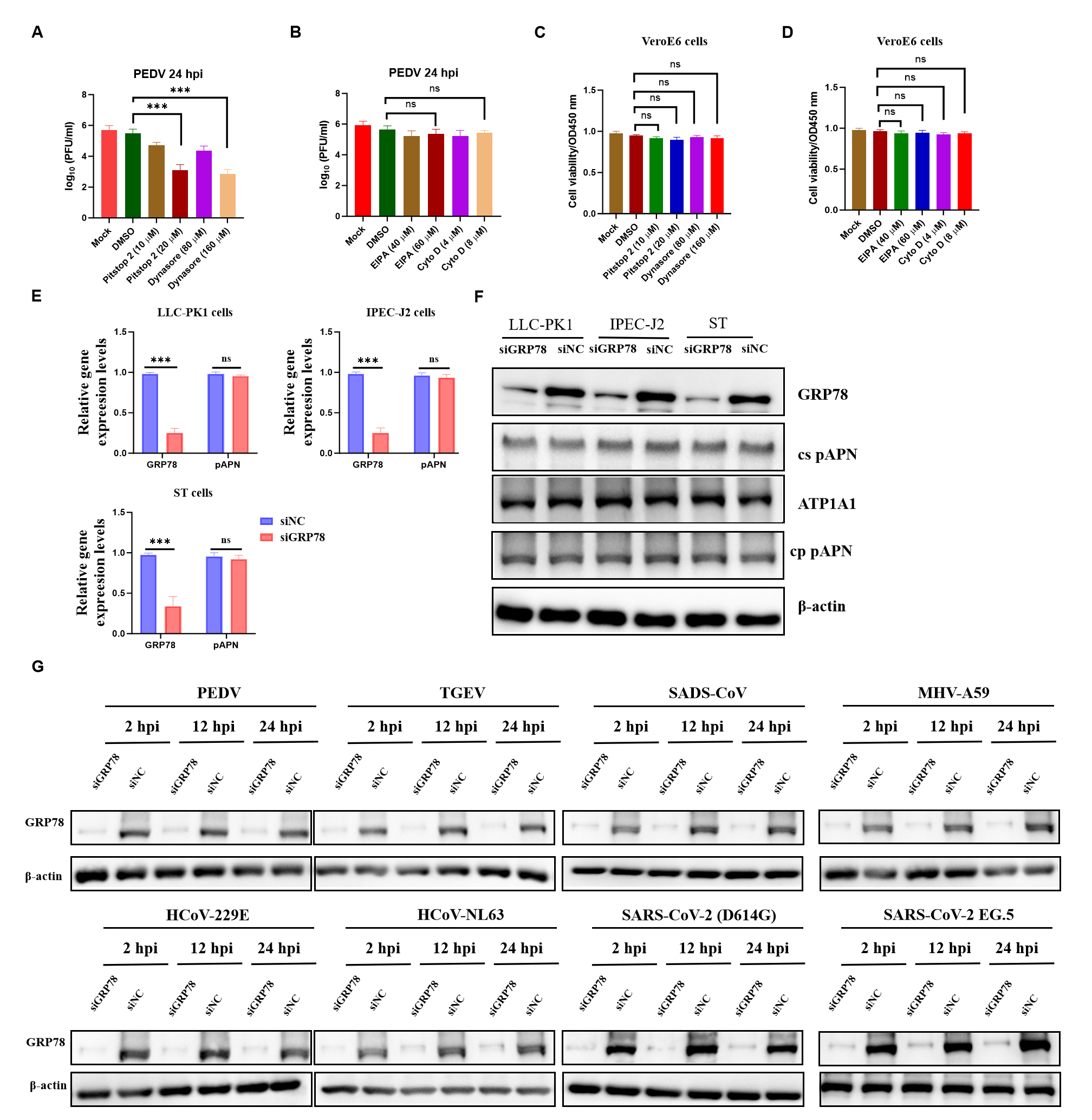

Supplement: Fig. S3 — Effects of clathrin/macropinocytosis inhibitors on PEDV infection and GRP78 knockdown on pAPN/GRP78 expression. [file jvi.00717-26-s0003.tif]
