# Supplementary material for: Identification of GRP78 as a novel host factor that facilitates zoonotic porcine deltacoronavirus internalization and replication via clathrin-mediated endocytosis
Source: J Virol. 2026 Jul 2;100(7):e00717-26. doi: 10.1128/jvi.00717-26 (PMC13386991; doi:10.1128/jvi.00717-26)
Supplement: Supplemental legends — Descriptive legends for supplemental material. [file jvi.00717-26-s0004.docx]

Supplemental Materials

Figure S1. Detection of target gene expression knockdown or knockout. (A) and (B): Knockdown efficiency of siRNAs targeting two genes simultaneously, as measured by WB and real-time RT-PCR, respectively. (C) Western blot analysis of target gene expression in HSPG2^KO^, pAPN^KO^, and HSPG2/pAPN^DKO^ IPEC-J2 cell lines. (D) and (E) Determination of the effect of GRP78 knockdown on pAPN protein expression levels or location in cells by RT-qPCR and Western blot (WB), respectively. Data are presented as means ± SD and are representative of at least two independent experiments. “ns” represents no significance, “*” represents p < 0.05, “**” represents p < 0.01, and “***” represents p < 0.001 (unpaired Student’s t-test).

Figure S2. Detection of cytotoxicity, antibody validation, GRP78 localization, and recombinant plasmid expression. (A) Detection of cytotoxicity of HA15 inhibitor on various experimental cell lines. (B) and (C)Validation of the PDCoV S1 monoclonal antibody by WB and IFA. (D) Detection of the GRP78 expression levels in the cell membrane and the cytoplasm using WB assay. (E) and (F) Determining the expression of the recombinant plasmid by IFA assay. Data are presented as means ± SD and are representative of at least two independent experiments. “ns” represents no significance and “***” represents p < 0.001 (unpaired Student’s t-test).

Figure S3. Effects of clathrin/macropinocytosis inhibitors on PEDV infection and GRP78 knockdown on pAPN/GRP78 expression. (A) Determination of the effects of clathrin inhibitors Pitstop 2 and Dynasore on PEDV infection titer by RT-qPCR. (B) Determination of the cytotoxicity of clathrin inhibitors Pitstop 2 and Dynasore on VeroE6 cells. (C) Determination of the effects of macropinocytosis inhibitors EIPA and Cyto D on PEDV infection titer by RT-qPCR. (D) Determination of the cytotoxicity of macropinocytosis inhibitors EIPA and Cyto D on VeroE6 cells. (E) Detection of GRP78 expression levels by Western blot (WB) after GRP78 knockdown in different cell lines. Data are presented as means ± SD and are representative of at least two independent experiments. “ns” represents no significance and “***” represents p < 0.001 (unpaired Student’s t-test).

Table S1. Results of LC-MS/MS analysis of host proteins co-immunoprecipitated with PDCoV S1.
